# ITC: Infused Tangential Curves for Smooth 2D and 3D Navigation of Mobile Robots †

**DOI:** 10.3390/s19204384

**Published:** 2019-10-10

**Authors:** Abhijeet Ravankar, Ankit A. Ravankar, Arpit Rawankar, Yohei Hoshino, Yukinori Kobayashi

**Affiliations:** 1 School of Regional Innovation and Social Design Engineering, Faculty of Engineering, Kitami Institute of Technology, Kitami, Hokkaido 090-8507, Japan; hoshinoy@mail.kitami-it.ac.jp; 2 Division of Human Mechanical Systems and Design, Faculty of Engg., Hokkaido University, Sapporo, Hokkaido 060-8628, Japan; ankit@eng.hokudai.ac.jp (A.A.R.); kobay@eng.hokudai.ac.jp (Y.K.); 3 Department of Electronics and Telecommunication, Vidyalankar Institute of Technology, Mumbai 400037, India; arpit.rawankar@vit.edu.in

**Keywords:** robot path smoothing, robot navigation, safe navigation, multi-robot navigation, collision avoidance

## Abstract

Navigation is an indispensable component of ground and aerial mobile robots. Although there is a plethora of path planning algorithms, most of them generate paths that are not smooth and have angular turns. In many cases, it is not feasible for the robots to execute these sharp turns, and a smooth trajectory is desired. We present ‘ITC: Infused Tangential Curves’ which can generate smooth trajectories for mobile robots. The main characteristics of the proposed ITC algorithm are: (1) The curves are tangential to the path, thus maintaining $G^{1}$ continuity, (2) The curves are infused in the original global path to smooth out the turns, (3) The straight segments of the global path are kept straight and only the sharp turns are smoothed, (4) Safety is embedded in the ITC trajectories and robots are guaranteed to maintain a safe distance from the obstacles, (5) The curvature of ITC curves can easily be controlled and smooth trajectories can be generated in real-time, (6) The ITC algorithm smooths the global path on a part-by-part basis thus local smoothing at one point does not affect the global path. We compare the proposed ITC algorithm with traditional interpolation based trajectory smoothing algorithms. Results show that, in case of mobile navigation in narrow corridors, ITC paths maintain a safe distance from both walls, and are easy to generate in real-time. We test the algorithm in complex scenarios to generate curves of different curvatures, while maintaining different safety thresholds from obstacles in vicinity. We mathematically discuss smooth trajectory generation for both 2D navigation of ground robots, and 3D navigation of aerial robots. We also test the algorithm in real environments with actual robots in a complex scenario of multi-robot collision avoidance. Results show that the ITC algorithm can be generated quickly and is suitable for real-world scenarios of collision avoidance in narrow corridors.

## 1. Introduction

It is well understood that, in the near future, mobile robots will replace many works currently done manually by people. These is mostly dull (moving stuff in warehouse), dangerous (handling hazardous materials), and demanding (lifting heavy items) work. In order ro set the scene for the paper, we take an example of a mobile item dispatch robot whose task is to carry items from one location to another location in an indoor environment. The environment has a lot of obstacles like furniture, walls, moving people, and other robots. The robot has a map of the environment in which the static obstacles and free navigational space are indicated. The robot must avoid collision with these static and dynamic obstacles. For this purpose, mobile robots have a path planning module to generate collision free trajectories from the start to the goal location.

Path planning is a two-step process [1]. The item dispatch robot would first plan a global path from the start to the goal location. At this stage, total path length is a dominating factor, and only the static obstacles are considered while making the path. Once the global path is generated, the item dispatch robot starts navigating on it. When the robot finds a dynamic obstacle, it should change its trajectory to avoid collision. This is done by the second stage of local planning. The local planner alters the trajectory to avoid collision with obstacles.

However, many path planners have a drawback that the path generated has sharp turns. This is undesired as it might be kinematically infeasible for the robot to execute these sharp turns without stopping or considerably reducing the speed. In addition, the robot’s maneuver on the paths with sharp turns is not natural for people in the vicinity, and they may fail to anticipate the robot’s path, which could lead to collision. A person walking behind the robot may dash into it if the robot suddenly stops to execute a sharp turn. Therefore, smooth paths are desired for robot motion. A robot can navigate the smooth paths at a constant velocity without completely stopping. The smooth paths are natural, and they are safe for the carried payload. Hence, path smoothing is important for safe robot navigation. Path smoothing is even more useful for applications like autonomous robotic wheelchairs to carry patients and disabled people.

We present an algorithm that can smooth the sharp turns of any traditional path planners while ensuring navigational safety. Our approach induces smooth curves thay are tangential to the original path. This tangentiality is important to enable robot navigation at a constant speed. It avoids any jerks or kinks in the path. We optimize the algorithm to generate these smooth induced curves for real-time applications. We mathematically formulate the problem and provide solutions for both 2D and 3D cases. The 2D case applies to UGVs (Unmanned Ground Vehicles), and the 3D case applies to UAVs (Unmanned Aerial Vehicles). We consider a complex distribution of obstacles and complicated maneuvers while discussing the proposed smoothing algorithm. In addition, we verify the accuracy and applicability of the proposed algorithm by providing results in both simulation and actual multi-robots in real environments, and comparing our results with the traditional algorithms.

Researchers have timely tested the existing path planning algorithms in various scenarios. Moreover, researchers have optimized these algorithms to meet the time constraints of real world applications. Therefore, the proposed work is not meant to replace already existing path planning algorithms. Instead, we propose the algorithm as a ‘smoothing extension‘ for already existing algorithms to smooth out the sharp turns.

This is an extension of our previous work [2]. We thoroughly improve the previous work through mathematical formations, proofs, safe navigation, generating trajectories of specific curvatures, more experiments in both simulation and real world scenarios, and a detailed analysis. We summarize the main characteristics and novel contributions of this work as follows:

1. The proposed ITC algorithm generates smooth curves that are tangential to the original path. Thus, $G^{1}$ continuity is always guaranteed.
2. The smooth curves are infused in the original global path to smooth out the turns. Thus, the original global path is kept intact.
3. The straight segments of the global path are kept straight and only the sharp turns are smoothed. This is advantageous to keep a safe distance from the walls while navigating a narrow corridor.
4. Safety is embedded in the ITC trajectories, and robots are guaranteed to maintain a safe distance from the obstacles.
5. The curvature of ITC curves can easily be controlled and smooth trajectories can be generated fast in real-time.
6. The ITC algorithm smooths the global path on a part-by-part basis thus local smoothing at one point does not affect the global path.
7. The path smoothing is possible for both 2D navigation of ground vehicles, and 3D navigation of aerial robots.
8. We present ITC as a smoothing extension that can work in conjunction with any of the traditional path planners.

### Related Works

The literature is full of global and local planning of mobile robots. The most widely used algorithms for global planning are: A* [3], D* [4,5], potential fields [6], Probabilistic Roadmap Planner (PRM) [7], rapidly exploring random tree (RRT) [8,9,10], and Dijkstra‘s algorithm [11,12,13], among many others. For local planning, the TEB (Timed-Elastic Band) planner [14] is worth mentioning. Many algorithms for cooperative multi-robot path planning have also been proposed [15,16]. Obstacle avoidance is an integral part of path planning and various approaches using visibility binary trees [17], inter-robot communication [18,19], path sharing [20], caching [21], and bio-inspired algorithms [22,23] have been proposed.

In recent years, with the proliferation of mobile robots and middle-ware robotics software like ROS (Robot Operating System), path smoothing has gained attention. Over the years, researchers have proposed to use geometric curves for smoothing paths. This includes approaches using clothoids [24,25], circle [26,27,28,29], splines (B-spline [30], intrinsic splines [31], quintic $G^{2}$ splines [32], non-uniform rational B-spline [33,34,35]), Bezier curve [36,37,38], hypocycloid [39,40], and other geometric curves. Path smoothing using interpolation [41] is also popular. Other researchers have used optimization techniques [42,43,44,45,46,47] to smooth robots’ paths. Among optimization based planners, ‘Time Elastic Band’ planner (TEB Planner) [14,48,49,50,51] is widely used. For an extensive review of various path smoothing algorithms, we direct the readers to a review of previous works related to path smoothing [1] that extensively compares the merits, demerits, and challenges of the various path smoothing approaches.

A map is a prerequisite for path planning. A map is generated by using any of the SLAM (Simultaneous Localization and Mapping) algorithms available in the literature (see [52,53,54]). A SLAM module basically builds a map of the environment, and simultaneously localizes the robot in it. However, if the map has already been generated, it can be reused and the robot just needs to localize itself in it by matching sensor data using different algorithms [52,55]. Most commonly used sensors are Lidar, camera, and inertial sensors like IMU (Inertial Measurement Units). In this paper, it is assumed that there are sensors attached to the robot to avoid obstacles, and localize the robot in the map that has been generated already.

## 2. Induced Tangential Curves: 2D Path Smoothing Case

This section discusses the path smoothing for the 2D case. The 3D path smoothing case is discussed in Section 5.

**Figure 1:**
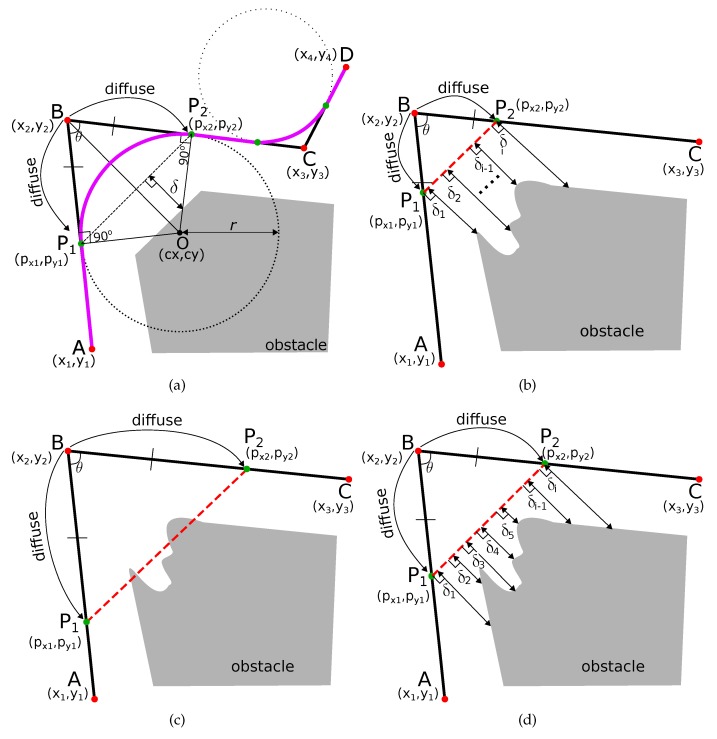
Diffusion in ITC (Infused Tangential Curves) based smoothing. (**a**) ITC curve $\overset{⏜}{P_{1}P_{2}}$ is induced in the original path and tangential at points $P_{1}$ and $P_{2}$; (**b**) under diffusion of point B; (**c**) over diffusion of point B; (**d**) appropriate diffusion of point B.

Most of the traditional path planning algorithms generate a path which has many sharp and angular turns. Let us assume that the path ABCD (shown in black in Figure 1) is a section of the robot’s path with a sharp turn at point B, and point C. The coordinates of the point A ($x_{1},y_{1}$), B ($x_{2},y_{2}$), C ($x_{3},y_{3}$), and D ($x_{4},y_{4}$) are also shown in Figure 1a. The obstacle is shown in gray color. The sharp turns at points B and C needs to be smoothed out.

First, we find two points $P_{1}$ and $P_{2}$ such that the line joining the two points $\bar{P_{1}P_{2}}$ is at a safe threshold distance ($\delta_{\mathrm{thresh}}$) from the obstacle. To find these points, we find intermediate points $P_{1}$ on the line $\bar{\mathrm{BA}}$ at a fixed small distance from the point B. We fix another intermediate point $P_{2}$ on the line $\bar{\mathrm{BC}}$ at the same fixed distance from the point B, i.e., $\bar{{\mathrm{BP}}_{1}}$ = $\bar{{\mathrm{BP}}_{2}}$. We define this process of finding intermediate points from the turn-point as ‘diffusion’. The turn point B is diffused into points $P_{1}$ and $P_{2}$ along the directions $\bar{\mathrm{BA}}$ and $\bar{\mathrm{BC}}$, respectively.

The turn point B is diffused into points $P_{1}$ and $P_{2}$ along the directions $\bar{\mathrm{BA}}$ and $\bar{\mathrm{BC}}$, such that the line joining the two points $\bar{P_{1}P_{2}}$ is at a safe threshold distance ($\delta_{\mathrm{thresh}}$) from the obstacle. If $\chi =\{\delta_{1},\delta_{2},\cdots ,\delta_{n}\}$ is a set of perpendicular distances from line $P_{1}P_{2}$ to the obstacle at different points on the line, then there are three cases for such diffusion.

1. **Under Diffusion**: This case is shown in Figure 1b. The line $\bar{P_{1}P_{2}}$ is too far from the obstacles and the minimum perpendicular distance from the line is greater than the minimum safety threshold distance, or $\min_{\delta_{i}\in \chi }>\delta_{\mathrm{thresh}}.$
2. **Over Diffusion**: This case is shown in Figure 1c. The line $\bar{P_{1}P_{2}}$ cuts through the obstacle.
3. **Appropriate Diffusion**: This case is shown in Figure 1d. The line $\bar{P_{1}P_{2}}$ is at an appropriate distance $\delta_{\mathrm{thresh}}$ from the obstacle. To facilitate programming, we define an ϵ
  (1) $$\min_{\delta_{i}\in \chi }=\delta_{\mathrm{thresh}}\pm \epsilon .$$
  The parameter ϵ enables adjusting the threshold distance considering the width of the robot.

**Figure 2:**
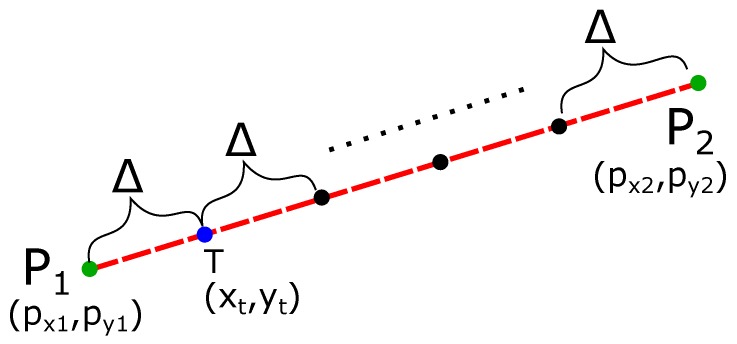
Discretizing the line $\bar{P_{1}P_{2}}$ into points $x_{t}^{i},y_{t}^{i}$ separated by Δ distance.

To find the minimum distance of the line $\bar{P_{1}P_{2}}$ from the obstacle ($\min_{\delta_{i}\in \chi }$), we discretize the line $\bar{P_{1}P_{2}}$ into several intermediate points ($x_{t},y_{t}$) separated by a small distance Δ, as shown in Figure 2. We then estimate the distance of the obstacle from each intermediate point ($x_{t}^{i},y_{t}^{i}$). The total points are

(2) $$n_{\mathrm{total}\_\mathrm{pts}}=\frac{\sqrt{{\left(p_{x2}-p_{x1}\right)}^{2}+{\left(p_{y2}-p_{y1}\right)}^{2}}}{\Delta }.$$

To find the intermediate point ‘T’ ($x_{t}^{i},y_{t}^{i}$) at the distance Δ from $P_{1}$, we first find the unit vector ($\hat{u}$) from the point $P_{1}$ to $P_{2}$. The unit vector is

(3) $$\hat{u}=\frac{p_{x2}-p_{x1}}{\sqrt{{\left(p_{x2}-p_{x1}\right)}^{2}+{\left(p_{y2}-p_{y1}\right)}^{2}}}\hat{x}+\frac{p_{y2}-p_{y1}}{\sqrt{{\left(p_{x2}-p_{x1}\right)}^{2}+{\left(p_{y2}-p_{y1}\right)}^{2}}}\hat{y},$$

where $\hat{x}$ and $\hat{y}$ are the unit vectors in the x and y directions. Point $T(x_{t}^{i},y_{t}^{i})$ at a distance Δ from the point $P_{1}$ along the line $\bar{P_{1}P_{2}}$ is

(4) $$\vec{T}=\vec{P_{1}}+d\hat{u}.$$

Splitting up the respective x and y components gives

(5) $$\begin{array}{c} x_{t}=p_{x1}+\frac{\Delta }{\sqrt{{\left(p_{x2}-p_{x1}\right)}^{2}+{\left(p_{y2}-p_{y1}\right)}^{2}}}\left(p_{x2}-p_{x1}\right),\\ t_{t}=p_{y1}+\frac{\Delta }{\sqrt{{\left(p_{x2}-p_{x1}\right)}^{2}+{\left(p_{y2}-p_{y1}\right)}^{2}}}\left(p_{y2}-p_{y1}\right). \end{array}$$

Thus, the intermediate points are calculated as

(6) $$\begin{array}{c} x_{t}^{i}=p_{x1}+\frac{\Delta \;\cdot \;i}{\sqrt{{\left(p_{x2}-p_{x1}\right)}^{2}+{\left(p_{y2}-p_{y1}\right)}^{2}}}\left(p_{x2}-p_{x1}\right),\;\;\;\;\;i\in \left\{1,2,\cdots ,n\right\}\;\;\mathrm{and}\;\;x_{t}^{i}\leq p_{x2},\\ y_{t}^{i}=p_{y1}+\frac{\Delta \;\cdot \;i}{\sqrt{{\left(p_{x2}-p_{x1}\right)}^{2}+{\left(p_{y2}-p_{y1}\right)}^{2}}}\left(p_{y2}-p_{y1}\right),\;\;\;\;\;i\in \left\{1,2,\cdots ,n\right\}\;\;\mathrm{and}\;\;y_{t}^{i}\leq p_{y2}. \end{array}$$

We briefly explain the calculation of the minimum distance $\min_{\delta_{i}\in \chi }$ from each intermediate point ($x_{t}^{i},y_{t}^{i}$). The slope of the line $\bar{P_{1}P_{2}}$ is

(7) $$m_{p_{1}p_{2}}=\frac{p_{y2}-p_{y1}}{p_{x2}-p_{x1}}.$$

The slope of line perpendicular to the line $\bar{P_{1}P_{2}}$ is

(8) $$m_{⊥}=\frac{-1}{m_{p_{1}p_{2}}}.$$

Using Equation (6), we generate points on the line from each point ($x_{t}^{i},y_{t}^{i}$) with slope $m_{⊥}$ in small units until the point touches the obstacle. Since the process is the same as explained above, we omit the explanation as it is straightforward.

Notice that Equation (6) is also used to diffuse the turn point B into intermediate points $P_{1}$ and $P_{2}$ along the directions $\bar{\mathrm{BA}}$ and $\bar{\mathrm{BC}}$ by the same distance Δ. Diffusion is stopped when appropriate points are found within the safe distance from the obstacle.

The two diffused points acts are points of contact of an induced smooth curve that is tangential at these points. In order to find the curve, we first find the circle and its radius whose arc will replace the angular path. The center of this circle is the point of intersection of the two perpendicular segments from these diffused points $P_{1}$ and $P_{2}$. The steps are explained below.

The slope of line $\bar{\mathrm{AB}}$ in Figure 1a is

(9) $$m_{AB}=\frac{y_{2}-y_{1}}{x_{2}-x_{1}},$$

and the slope of line $\bar{\mathrm{BC}}$ in Figure 1a is

(10) $$m_{BC}=\frac{y_{2}-y_{3}}{x_{2}-x_{3}}.$$

In Figure 1a, the line $\bar{P_{1}O}$ is perpendicular to the line $\bar{\mathrm{AB}}$ from point $P_{1}\left(p_{x1},p_{y1}\right)$. Hence, the slope of line $\bar{P_{1}O}$ is

(11) $$m_{P_{1}O}=\frac{-1}{m_{AB}}.$$

Similarly, line $\bar{P_{2}O}$ is perpendicular to the line $\bar{\mathrm{BC}}$ from point $P_{2}\left(p_{x2},p_{y2}\right)$, and the slope of line $\bar{P_{2}O}$ is

(12) $$m_{P_{2}O}=\frac{-1}{m_{BC}}.$$

The point of intersection of lines $\bar{P_{1}O}$ and $\bar{P_{2}O}$ (point $O(c_{x},c_{y})$ in Figure 1a) is the center of the circle whose arc will define the smoothed path.

The general equation of a line of slope *m* passing through a point $\left(x_{1},y_{1}\right)$ is

(13) $$\begin{array}{c} y-y_{1}=m\left(x-x_{1}\right)\\ \Rightarrow y=mx-mx_{1}+y_{1}. \end{array}$$

Since the lines $\bar{P_{1}O}$ and $\bar{P_{2}O}$ intersect at $O(c_{x},c_{y})$,

$$\begin{array}{cc} c_{y} & =m_{P_{1}O}\left(c_{x}-p_{x1}\right)+p_{y1}=m_{P_{2}O}\left(c_{x}-p_{x2}\right)+p_{y2},\\ \; & \Rightarrow m_{P_{1}O}\;\cdot \;c_{x}-m_{P_{1}O}\;\cdot \;p_{x1}+p_{y1}=m_{P_{2}O}\;\cdot \;c_{x}-m_{P_{2}O}\;\cdot \;p_{x2}+p_{y2}\\ \; & \Rightarrow m_{P_{1}O}\;\cdot \;c_{x}-m_{P_{2}O}\;\cdot \;c_{x}=m_{P_{1}O}\;\cdot \;p_{x1}-m_{P_{2}O}\;\cdot \;p_{x2}+p_{y2}-p_{y1}\\ \; & \Rightarrow c_{x}\;\cdot \;\left(m_{P_{1}O}-m_{P_{2}O}\right)=m_{P_{1}O}\;\cdot \;p_{x1}-m_{P_{2}O}\;\cdot \;p_{x2}+p_{y2}-p_{y1}. \end{array}$$

Thus, the *x*-coordinate of the center of the circle is

(14) $$c_{x}=\frac{m_{P_{1}O}\;\cdot \;p_{x1}-m_{P_{2}O}\;\cdot \;p_{x2}+p_{y2}-p_{y1}}{\left(m_{P_{1}O}-m_{P_{2}O}\right)}.$$

The *y*-coordinate of the center of the circle can be obtained by plugging the value of $c_{x}$ in the equation of line $\bar{P_{1}O}$ or $\bar{P_{2}O}$,

(15) $$c_{y}=m_{P_{1}O}\;\cdot \;c_{x}-m_{P_{1}O}\;\cdot \;p_{x1}+p_{y1}.$$

The radius of the circle *r* is

(16) $$r=\sqrt{{\left(c_{x}-p_{x1}\right)}^{2}+{\left(c_{y}-p_{y1}\right)}^{2}},\;\;\;\mathrm{or}\;\;\;r=\sqrt{{\left(c_{x}-p_{x2}\right)}^{2}+{\left(c_{y}-p_{y2}\right)}^{2}}.$$

The circle with center ($c_{x},c_{y}$) and radius *r* is shown in Figure 1a. We take the arc $\overset{⏜}{P_{1}P_{2}}$ of the circle between points $P_{1}$ and $P_{2}$ shown in magenta in Figure 1a. This curve $\overset{⏜}{P_{1}P_{2}}$ has the following properties:

- Curve $\overset{⏜}{P_{1}P_{2}}$ is tangential to the robot’s original path. Hence, $G^{1}$ geometric continuity is guaranteed.
- Curve $\overset{⏜}{P_{1}P_{2}}$ is smoother to traverse compared to the original path of the robot. In Figure 1a, the original path of the robot is ABC with sharp turn at the point B. The smooth path is $\overset{⏜}{{\mathrm{AP}}_{1}P_{2}C}$.
- Curve $\overset{⏜}{P_{1}P_{2}}$ is ‘infused’ inside the original path of the robot.

Due to the three properties above, $\overset{⏜}{P_{1}P_{2}}$ is called an ‘Infused Tangential Curve’.

## 3. Accelerating ITC Path Smoothing Algorithm

The smoothing algorithm is accelerated on two fronts. First, we accelerate the diffusion of the point of sharp turn. Second, we also accelerate the algorithm to estimate the minimum distance from the obstacles. These are explained below.

### 3.1. Accelerating the Diffusion Algorithm

**Figure 3:**
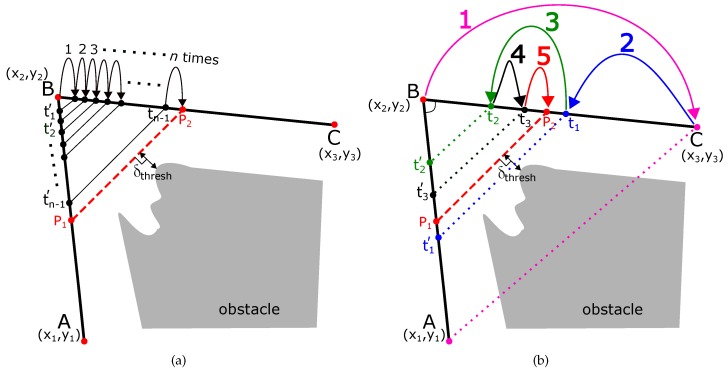
Normal and accelerated diffusion of point B. (**a**) normal diffusion; (**b**) accelerated diffusion based on binary search.

Figure 3a shows the normal approach of diffusing the node B ($x_{2},y_{2}$) into points $P_{1}$ and $P_{2}$. The normal algorithm diffuses the point into small increments of $\Delta_{\mathrm{diff}}$. As shown in Figure 3a, if $P_{1}$ ($p_{x1},p_{y1}$) and $P_{2}$ ($p_{x2},p_{y2}$) are the appropriate diffused positions which maintain a safe threshold distance ($\delta_{\mathrm{thresh}}$) from the obstacles, the total steps $n_{\mathrm{diff}}$ are

(17) $$n_{\mathrm{diff}}=\frac{\sqrt{{\left(p_{x2}-x_{2}\right)}^{2}+{\left(p_{y2}-y_{2}\right)}^{2}}}{\Delta_{\mathrm{diff}}}.$$

Hence, normal diffusion algorithm would have a complexity of $O(n_{\mathrm{diff}})$.

We accelerate the algorithm using binary search. The idea is shown in Figure 3b, and it is used for explanation. In the binary search, the first diffusion of point B ($x_{2},y_{2}$) occurs at the maximum distance at point C ($x_{3},y_{3}$). This is shown by a magenta-colored arrow in Figure 3b. A check is performed if the line joining the diffused points i.e., $\bar{\mathrm{AC}}$ maintains a safe threshold from the obstacles. As shown in Figure 3b, the line $\bar{\mathrm{AC}}$ crosses over the obstacle, and it is a case of ‘over-diffusion’. Therefore, in step 2, the diffusion distance is half the distance of the previous diffusion (i.e., $\frac{\sqrt{{\left(x_{3}-x_{2}\right)}^{2}+{\left(y_{3}-y_{2}\right)}^{2}}}{2}$). This is shown by a blue arrow in Figure 3b, and the safety threshold is checked again. This is again a case of over-diffusion, so, in step 3, the new diffusion distance is half the distance of the previous diffusion (i.e., $\frac{\sqrt{{\left(x_{3}-x_{2}\right)}^{2}+{\left(y_{3}-y_{2}\right)}^{2}}}{4}$) and shown with a green arrow. A check for safety clearance is performed, and it is determined to be a case of ‘under-diffusion’. Due to this, in the next step 4, the diffusion is increased by half of the previous distance (i.e., $\frac{3\;\cdot \;\sqrt{{\left(x_{3}-x_{2}\right)}^{2}+{\left(y_{3}-y_{2}\right)}^{2}}}{8}$). This process is repeated until the appropriate diffusion point ($P_{2}$) has been found.

Compared to the normal process, the accelerated algorithm takes $\log(n_{\mathrm{diff}})$ steps, and the complexity of the algorithm is $O(\log n_{\mathrm{diff}})$. The pseudo-code is given in Algorihtm 1.

**Algorithm 1:** Fast Algorithm to Find Diffusion Point

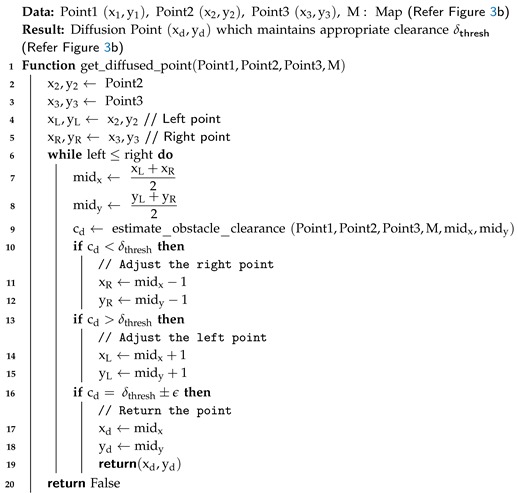



### 3.2. Accelerating the Minimum Distance Calculation

Similarly, we also accelerate the perpendicular distance estimation using the binary search algorithm.

**Figure 4:**
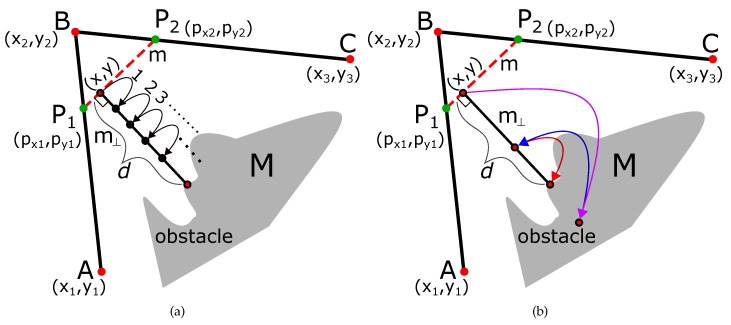
Normal and accelerated calculate of minimum distance from the obstacle. (**a**) normal approach; (**b**) accelerated approach.

Figure 4a shows the normal incremental approach of finding the perpendicular distance from the obstacle on the line with starting point (x,y) and slope $m_{⊥}$. The normal algorithm diffuses the point into small increments of $\Delta_{⊥}$. As shown in Figure 4a, the total steps $n_{\mathrm{clearance}}$ are

(18) $$n_{\mathrm{clearance}}=\frac{\mathrm{Perpendicular}\;\mathrm{distance}\;\mathrm{to}\;\mathrm{obstacle}}{\Delta_{⊥}}.$$

Hence, the algorithm has a complexity of $O(n_{\mathrm{clearance}})$.

The accelerated algorithm using binary search is explained in Figure 4. Since the idea is similar to that explained in the previous section, we omit explanation, and the complexity of the algorithm is $O(\log n_{\mathrm{clearance}})$. The pseudo-code is given in Algorithm 2.

The values of $n_{\mathrm{diff}}$, $n_{\mathrm{clearance}}$, and $n_{\mathrm{total}\_\mathrm{pts}}$ are given in Equations (2), (17), and (18), respectively. Compared to the incremental algorithm, the overall speedup is

(19) $$\mathrm{speedup}=\frac{n_{\mathrm{diff}}\times n_{\mathrm{clearance}}\times n_{\mathrm{total}\_\mathrm{pts}}}{\log\left(n_{\mathrm{diff}}\right)\times \log\left(n_{\mathrm{clearance}}\right)\times n_{\mathrm{total}\_\mathrm{pts}}}.$$

**Algorithm 2:** Estimate Obstacle Clearance

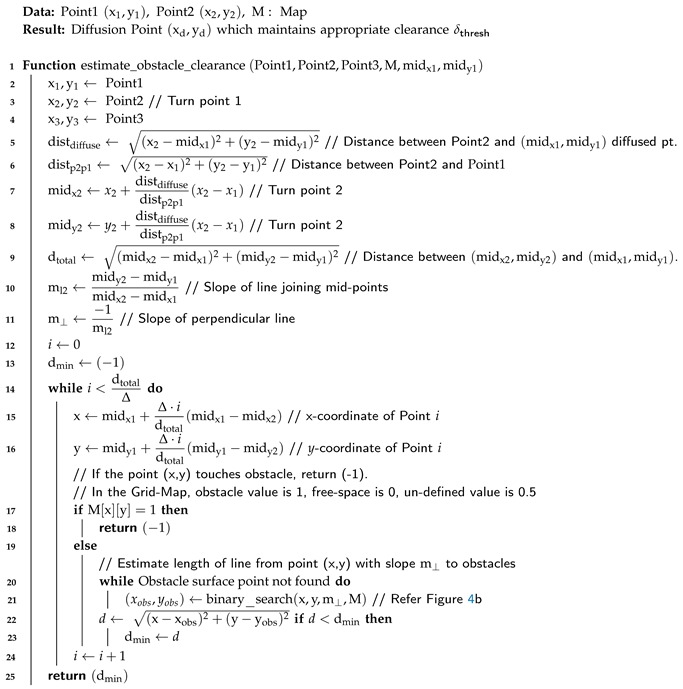



## 4. Robot’s Proximity from Obstacles on the Smooth Path

**Figure 5:**
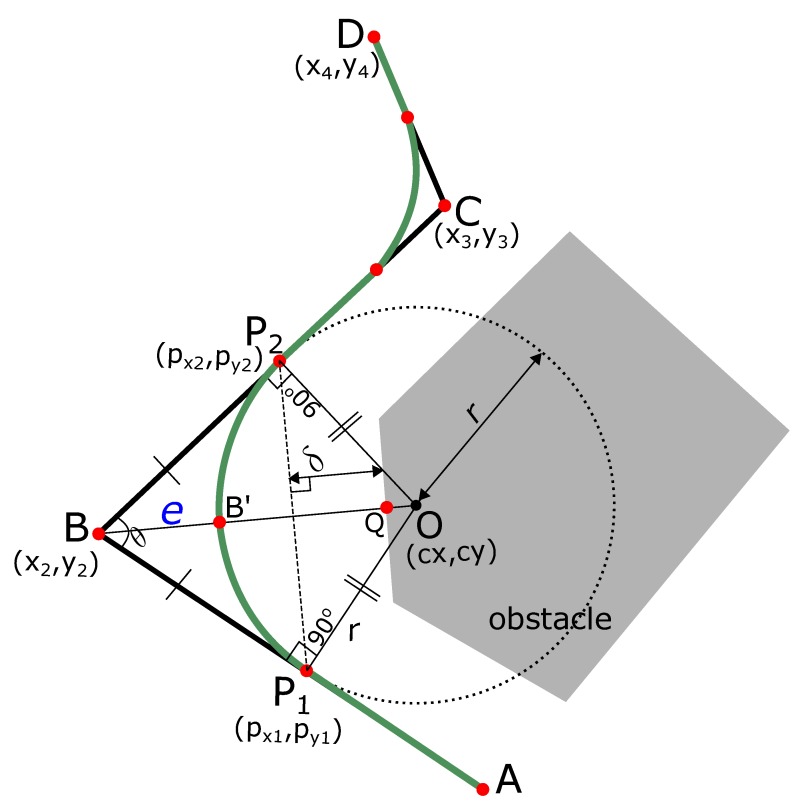
Calculation of robot’s proximity from obstacles on the smooth path compared to the original path.

In Figure 5, the original path ABCD of the robot with sharp turns at points B and C has been smoothed by the path $\overset{⏜}{{\mathrm{AP}}_{1}P_{2}}$. Originally, the robot would make a turn at point B keeping a distance BQ from the obstacle. On the smooth path, the robot comes closer to the obstacle while traversing the smooth green curve, and the distance is $B^{\prime }Q$. The proximity difference (shown as *e* in Figure 5) is $e={\mathrm{BB}}^{\prime }$. Since the robot itself has some width, the overall proximity to the obstacle will be increased while traversing the smooth curve. Hence, it is important to calculate this difference in proximity.

In Figure 5, we calculate the ∠ABC=θ using the cosine law as

(20) $$\begin{array}{cc} \theta & =\cos^{-1}\left(\frac{a^{2}+c^{2}-b^{2}}{2ac}\right),\\ \; & \mathrm{where},\\ a & =\sqrt{{\left(p_{x2}-x_{2}\right)}^{2}+{\left(p_{y2}-y_{2}\right)}^{2}},\\ b & =\sqrt{{\left(p_{x2}-p_{x1}\right)}^{2}+{\left(p_{y2}-p_{y1}\right)}^{2}},\\ c & =\sqrt{{\left(p_{x1}-x_{2}\right)}^{2}+{\left(p_{y1}-y_{2}\right)}^{2}}. \end{array}$$

In $△P_{1}OB$ and $△P_{2}OB$, we have

(21) $$\begin{array}{cc} \; & P_{2}B=P_{1}B\;\;(\mathrm{diffusion}\;\mathrm{by}\;\mathrm{same}\;\mathrm{distance})\\ \; & P_{2}O=P_{1}O\;\;(\mathrm{circle}\;\mathrm{radius}\;‘r’)\\ \; & BO=BO\;\;(\mathrm{common}\;\mathrm{side})\\ ∴ & △P_{1}OB≅△P_{2}OB\;\;(\mathrm{Congruent}\;\mathrm{triangles}\;\mathrm{by}\;\mathrm{SSS}\;\mathrm{Congruence}\;\mathrm{Theorem})\\ ∴ & ∠P_{1}BO=∠P_{2}BO\;\;(\mathrm{From}\;\mathrm{property}\;\mathrm{of}\;\mathrm{congruent}\;\mathrm{triangles})\\ \; & ∠P_{1}BO+∠P_{2}BO=\theta \;\;\left(\mathrm{Figure}5\right)\\ ∴ & ∠P_{1}BO=∠P_{2}BO=\frac{\theta }{2}\;\;\left(\mathrm{Figure}5\right). \end{array}$$

In other words, $\bar{BO}$ bisects $∠P_{1}BP_{2}$, and $∠P_{1}BO=\frac{\theta }{2}$. In $△P_{1}OB$, point $P_{1}$ is the tangent point, therefore $∠BP_{1}O=90^{\circ }$. Therefore,

(22) $$\begin{array}{c} \sin\frac{\theta }{2}=\frac{P_{1}O}{BO}=\frac{P_{1}O}{BB^{\prime }+B^{\prime }O}=\frac{r}{e+r}. \end{array}$$

Hence, the distance $e=\bar{BB^{\prime }}$ is calculated as

(23) $$\begin{array}{cc} \; & e=\frac{r}{\sin\frac{\theta }{2}}-r,\\ \mathrm{or},\; & e=r(\cos\mathrm{ec}\frac{\theta }{2}-1). \end{array}$$

Assuming that the robot has a width of $W_{r}$ and it accurately traverses the curve, the proximity to the obstacles is increased by a distance $\frac{W_{r}}{2}$. If the proximity of the robot is less than the threshold distance $\delta_{\mathrm{thresh}}$, the diffusion points $P_{1}$ and $P_{2}$ are moved closer to the point B by a distance of $\frac{W_{r}}{2}$, and the curve is recomputed to ensure safety.

## 5. Induced Tangential Curves: 3D Path Smoothing Case

We now explain the case of 3D path smoothing for UAVs (Unmanned Aerial Vehicles) and drones. The overall idea for the 3D case is an extension of the 2D case. ‘Node’ or the point of turn is now a point in 3D space. First, the overall path is generated by using traditional path planners. Commonly used robot path planning algorithms like A* algorithm [3], D* algorithm [4,5], potential fields algorithm [6], Probabilistic Roadmap Planner (PRM) [7], rapidly exploring random tree algorithm (RRT) [8,9,10], etc. generally have 3D extensions. Hence, any of the traditional algorithms can be used to generate the overall path.

**Figure 6:**
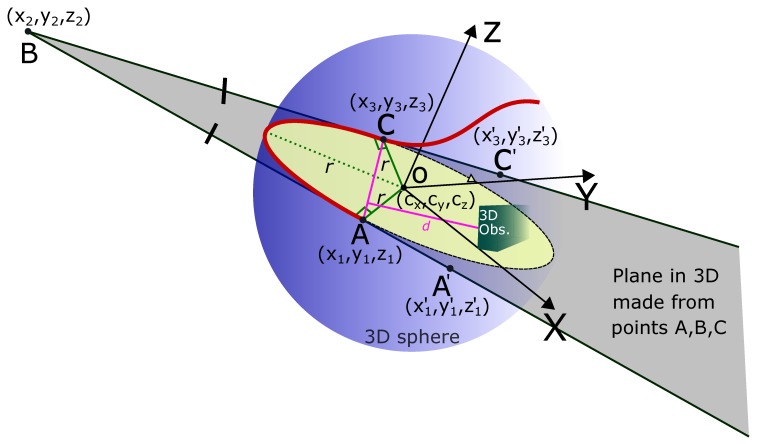
3D path smoothing with ITC. The points A,B, and C are in 3D space. The intersection of the 3D sphere and the plane generated from the three points marks the arc to smooth the path.

Figure 6 shows three points $A^{\prime }$$\left(x_{1}^{{}^{\prime }},y_{1}^{{}^{\prime }},z_{1}^{{}^{\prime }}\right)$, B$\left(x_{2},y_{2},z_{2}\right)$, and $C^{\prime }$$\left(x_{3}^{{}^{\prime }},y_{3}^{{}^{\prime }},z_{3}^{{}^{\prime }}\right)$ in 3D space. These points mark the original path of the robot. Point B is the point with sharp turn on the robot’s path. As explained in the previous sections, point B has been diffused to points A and C which are equidistant from point B, i.e., |AB|=|BC|. The line joining the two 3D points $\bar{\mathrm{AC}}$ is at a safe distance ($d=\delta_{\mathrm{thresh}}$) from the obstacle in 3D space shown in green in Figure 6. The aim is to find the circle (shown in yellow in Figure 6).

The yellow circle in Figure 6 lies on the plane which is formed by the three points A$\left(x_{1},y_{1},z_{1}\right)$, B $\left(x_{2},y_{2},z_{2}\right)$, and C$\left(x_{3},y_{3},z_{3}\right)$. The plane is shown in gray color.

The equation of the plane formed by three points is

(24) Ax+By+Cz+D=0,

where

$$A=\left[\begin{array}{ccc} 1 & y_{1} & z_{1}\\ 1 & y_{2} & z_{2}\\ 1 & y_{3} & z_{3} \end{array}\right],B=\left|\begin{array}{ccc} x_{1} & 1 & z_{1}\\ x_{2} & 1 & z_{2}\\ x_{3} & 1 & z_{3} \end{array}\right|,C=\left[\begin{array}{ccc} x_{1} & y_{1} & 1\\ x_{2} & y_{2} & 1\\ x_{3} & y_{3} & 1 \end{array}\right]$$

and

$$D=\left[\begin{array}{ccc} x_{1} & y_{1} & z_{1}\\ x_{2} & y_{2} & z_{2}\\ x_{3} & y_{3} & z_{3} \end{array}\right].$$

The equation of line AB and BC is given as

(25) $$\left(x_{1},y_{1},z_{1}\right)+t\left(x_{2}-x_{1},y_{2}-y_{1},z_{2}-z_{1}\right),$$

and

(26) $$\left(x_{2},y_{2},z_{2}\right)+t\left(x_{3}-x_{2},y_{3}-y_{2},z_{3}-z_{2}\right),$$

respectively.

The normal of the plane is calculated by taking the cross product,

(27) n=(A−B)×(C−B).

*n* is the vector (A,B,C) formed by three of the coefficients from the equation of the plane in Equation (24). The direction within the plane and perpendicular to AB is

(28) v=n×(A−B).

Similarly, the direction within the plane perpendicular to BC is

(29) w=n×(C−B).

A point on these lines can be represented as

(30) A+λvandC+μw.

The equation of a point which lies on both of them is

(31) D=A+λv=C+μw.

We solve for λ and μ. There are actually three equations, one for each coordinate, and two variables, so the system is over-determined. We can use pseudo inverse to avoid special cases and get the center of the sphere. Once we have the center, we can calculate the radius (*r*) of the circle with center $O\;(c_{x},c_{y},c_{z})$,

(32) $$r=\sqrt{{\left(c_{x}-x_{2}\right)}^{2}+{\left(c_{y}-y_{2}\right)}^{2}+{\left(c_{z}-z_{2}\right)}^{2}}.$$

As shown in Figure 6, the plane defined from the points A, B, and C cuts through the blue sphere. The boundary of the intersection of the sphere and the plane is a circle shown in yellow in Figure 6. The circle can be described with a parametric description, for which we require two orthonormal vectors within the plane. One such vector is

(33) $$e_{1}=\frac{\left(A-B\right)}{∥A-B∥},$$

and the other is

(34) $$e_{2}=\frac{\left(n\times e_{1}\right)}{∥n\times e_{1}∥}.$$

Once the circle has been calculated, we take the arc $\overset{⏜}{\mathrm{AC}}$ to generate the smooth trajectory. The original path of the robot was $A^{\prime }{\mathrm{BC}}^{\prime }$. The smooth path is $\overset{⏜}{A^{\prime }{\mathrm{ACC}}^{\prime }}$.

## 6. Experiments and Results

We now discuss the results of path smoothing using the proposed ITC algorithm. Section 6.1 first shows the 2D results. The 3D path smoothing results are shown in Section 6.2. Section 6.3 discusses the comparison with interpolation based smoothing algorithms. Section 6.4 shows results of smooth path generation with different curvatures. Section 6.5 shows smoothing results with actual robots in a real environment while discussing a multi-robot collision avoidance scenario.

### 6.1. Results of 2D Path Smoothing

**Figure 7:**
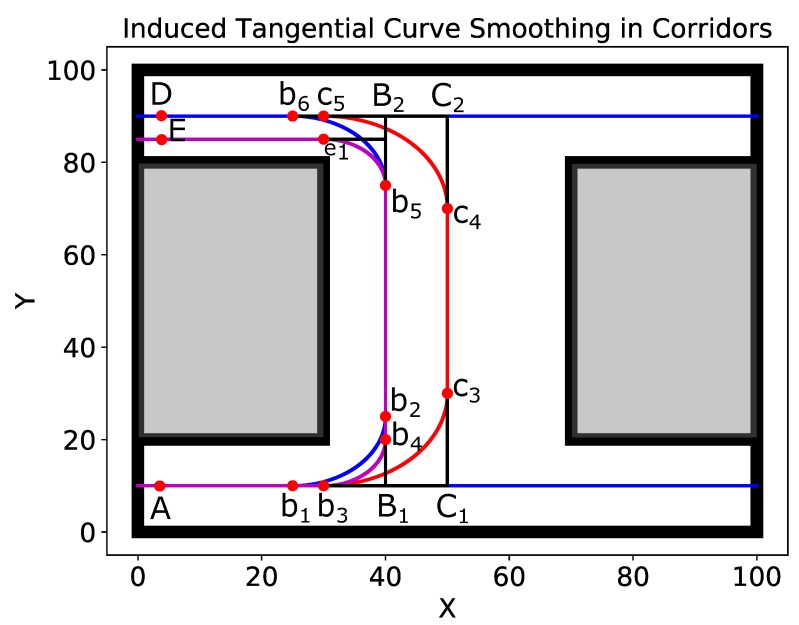
The case of path smoothing in corridors using ITC.

We first discuss path smoothing in indoor corridor environments which are frequently navigated by mobile service robots. Such an environment is shown in Figure 7 in which the smooth paths are also shown. In such environments, robots generally navigate the center of the corridor maintaining a safe distance from the walls on both sides. On the other hand, some robots are programmed to navigate the corridors on the left or right side. We discuss these cases below.

- Case I: As shown in Figure 7, if the robot is programmed to navigate the center of the corridors, the original path of the robot is ${\mathrm{AC}}_{1}C_{2}D$ with A as the starting point and D as the goal point.
  The path has two 90-degree turns at points $C_{1}$ and $C_{2}$. Two smooth curves $\overset{⏜}{b_{3}c_{3}}$ and $\overset{⏜}{c_{4}c_{5}}$ have been induced in the original path. The smooth path is $\overset{⏜}{{\mathrm{Ab}}_{3}c_{3}c_{4}c_{5}D}$.
- Case II: For ‘left-traversal’ robots, the original path of the robot is ${\mathrm{AB}}_{1}B_{2}D$ with A as the starting point and D as the goal point. This path also has two 90-degree turns at points $B_{1}$ and $B_{2}$. Two smooth curves $\overset{⏜}{b_{1}b_{2}}$ and $\overset{⏜}{b_{5}b_{6}}$ have been induced in the original path. The smooth path is $\overset{⏜}{{\mathrm{Ab}}_{1}b_{2}b_{5}b_{6}D}$.
  Two other curves $\overset{⏜}{b_{3}b_{4}}$ and $\overset{⏜}{b_{5}e_{1}}$ have also been induced in the original path for different curvature requirements. In any case, all of the induced curves are tangential and guarantee $G^{1}$ continuity.

**Figure 8:**
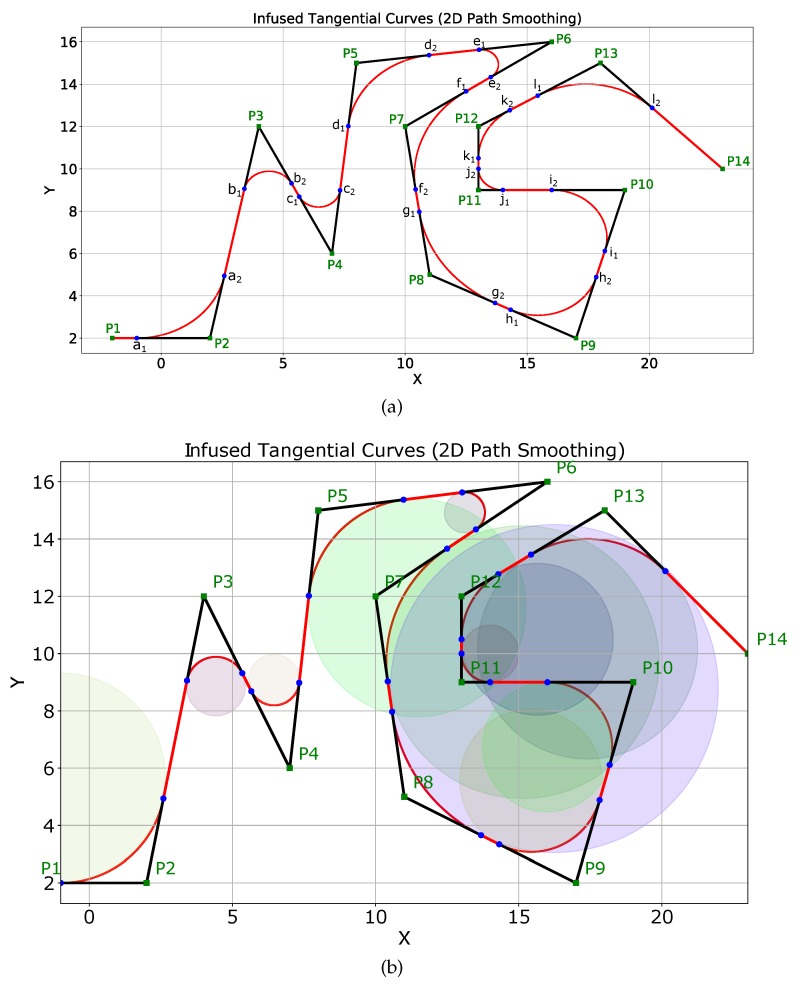
2D path smoothing using ITC in a complex scenario. (**a**) tangential curves $a_{1}a_{2},\;b_{1}b_{2},\;c_{1}c_{2},\;\cdots ,\;i_{1}i_{2}$ have been induced to smooth sharp turns with different $\delta_{\mathrm{thresh}}$; (**b**) visual representation of the different curves and their curvatures.

Figure 8a shows a complex scenario with many sharp turns of different angles. The original path of the robot is shown in black with the start point as P1 and the goal point as P14. There are sharp turns at points P2,P3, ⋯, P13. A major advantage of the proposed ITC algorithm is that it is possible to have a different safe threshold distance ($\delta_{\mathrm{thresh}}$) at different turn points based on several factors. In the simulation, we fixed different thresholds for different turn points as shown in Figure 8a. This is evident from the fact that the curvature of different induced curves ($\overset{⏜}{a_{1}a_{2}},\;\overset{⏜}{b_{1}b_{2}},\;\overset{⏜}{c_{1}c_{2}},\;\cdots ,\;\overset{⏜}{i_{1}i_{2}}$) shown in red in Figure 8a is different. For example, the curvature of the induced curve $\overset{⏜}{a_{1}a_{2}}$ is less than that of curve $\overset{⏜}{j_{1}j_{2}}$, as the safety threshold distance for the curve $\overset{⏜}{a_{1}a_{2}}$ ($\delta_{\mathrm{thresh}}=3$) was more than the safety threshold distance for the curve $\overset{⏜}{j_{1}j_{2}}$ ($\delta_{\mathrm{thresh}}=1$).

The curvatures of different curves can also be checked visually from Figure 8b, which shows the different circles whose segments are used for smoothing the turns. It is also visually evident that $\overset{⏜}{j_{1}j_{2}}$ and $\overset{⏜}{b_{1}b_{2}}$ are generated from arcs of circles with different radii, and hence different curvatures.

Table 1 summarizes the coordinates of the points and distance thresholds set for the different curves $\overset{⏜}{a_{1}a_{2}},\;\overset{⏜}{b_{1}b_{2}},\;\overset{⏜}{c_{1}c_{2}},\;\cdots ,\;\overset{⏜}{i_{1}i_{2}}$ shown in Figure 8a.

**Figure 9:**
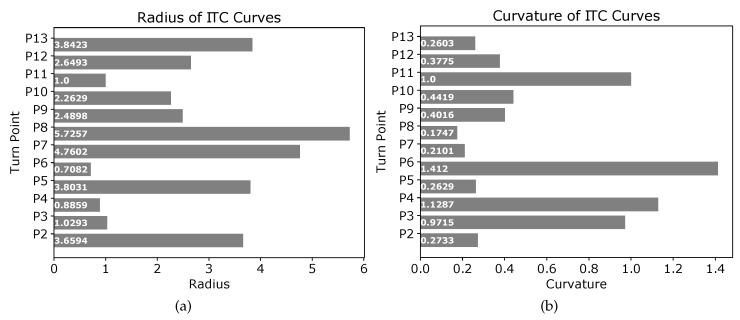
Radius and curvature of the different curves in Figure 8a. (**a**) radius of curves at different points; (**b**) curvature of curves at different points.

The threshold distances summarized in Table 1 should not be confused with the radius of the circles used to generate the tangential curves. The radii of different circles used to generate tangential curves at points P2,P3,⋯,P13 of Figure 8 are shown in Figure 9a. Similarly, the curvature of the different ITC curves are shown in Figure 9b. The ITC curve at point P6 ($\overset{⏜}{e_{1}e_{2}}$) had the minimum radius of 0.7082 units and thereby the minimum curvature, whereas the ITC curve at point P8 ($\overset{⏜}{g_{1}g_{2}}$) had the maximum radius and thereby the minimum curvature.

### 6.2. Results of 3D Path Smoothing

**Figure 10:**
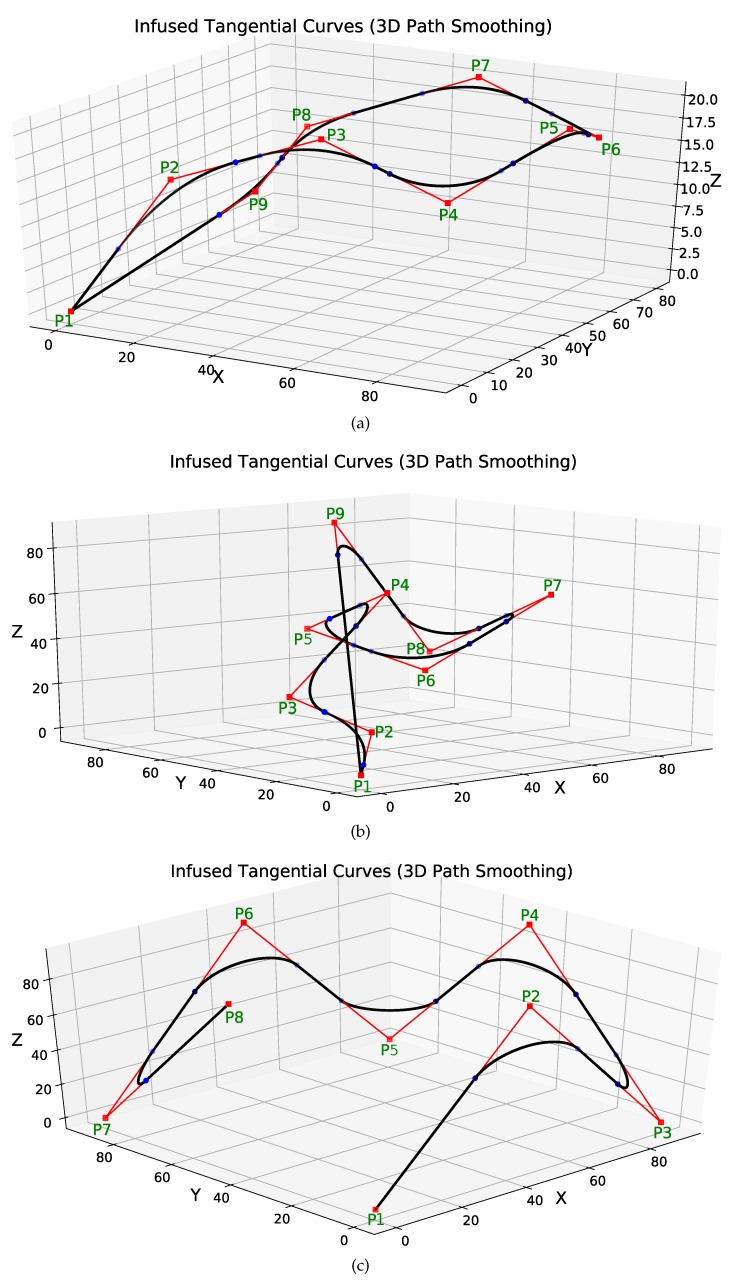
3D path smoothing. (**a**) closed loop 3D path smoothing; (**b**) a complex closed loop 3D path smoothing case; (**c**) an open loop 3D path smoothing case.

Figure 10 shows three complex scenarios of path smoothing for UAVs in 3D space. The original path of the UAV is shown in red. The smoothed paths are shown in black. The 3D points have been marked in green.

Figure 10a shows the first case of 3D path smoothing. The original path of the UAV is a closed-loop path with the same start and goal points (x=y=z=0). Different values of threshold distances have been used to generate smooth curves of appropriate curvatures. The 3D coordinates of different points and different clearance thresholds have been summarized in Table 2.

Similarly, Figure 10b also shows a closed path closed-loop UAV path with the same start and goal points (x=y=z=0). Compared to the path in Figure 10a, the path is more complex with difficult maneuvers and sharp turns. However, the proposed method is still able to smooth the sharp and angular turns. Different values of threshold distances have been used to generate smooth curves of appropriate curvatures. The 3D coordinates of different points and different clearance thresholds have been summarized in Table 3.

Figure 10c shows an open-loop UAV path with different start (P1:x=y=z=0) and goal points (P8:x=0,y=45,x=90). There are six sharp turns at points P2,P3,P4,⋯,P7. It is clear from Figure 10c that the proposed method keeps straight paths of the UAV straight. The turns can be smoothed for different curvatures. In this case, we used the same clearance threshold distance for the different turns. Hence, the curvature of all the smooth induced curves is the same. The 3D coordinates of different points and different clearance thresholds have been summarized in Table 4.

**Figure 11:**
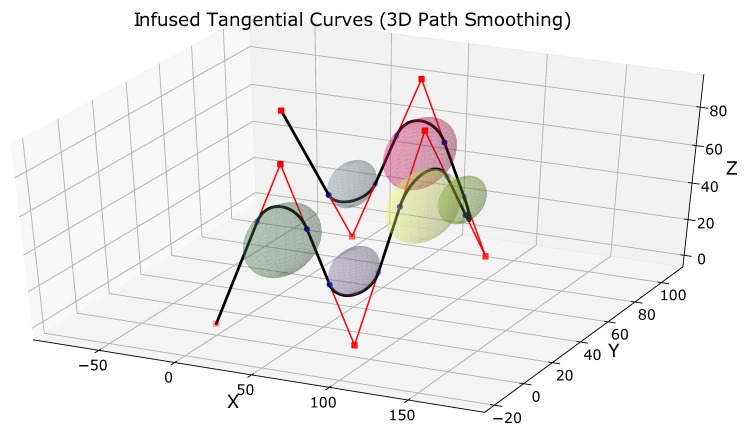
Visual representation of 3D path smoothing in case of Figure 10c.

Figure 11 shows the different 3D spheres for path smoothing in case of Figure 10c. The coordinates of the points are the same as given in Table 4. In this case, the surface of the 3D sphere is used to smooth out the sharp turns. It should be noted that the spheres in Figure 11 look like a circle as only a particular projection of the 3D space is shown.

### 6.3. Comparison with Other Works

In order to compare the strengths of our work, we compare our work with the path smoothing method proposed in the works of Huh and Chang in [56]. The method proposed in this paper uses an interpolation technique to smooth the sharp turns of the robot’s path. Interpolation technique was first proposed by Warning [57,58]. Precisely, given m+1 pairs $\left(x_{i},y_{i}\right)$, the problem consists of finding a function ϕ=ϕ(x) such that $\phi \left(x_{i}\right)=y_{i}$ for $i=0,\cdots ,m,y_{i}$ being some given values, and say that ϕ interpolates $\left\{y_{i}\right\}$ at the nodes $\left\{x_{i}\right\}$. We speak about polynomial interpolation if ϕ is an algebraic polynomial, trigonometric approximation if ϕ is a trigonometric polynomial, or piecewise polynomial interpolation (or spline interpolation) if ϕ is only locally a polynomial [1]. As interpolation based methods are widely used in path smoothing algorithms [41,59,60] found in the state-of-the-art, comparison with this method can highlight the merits as well as drawbacks of the proposed method.

**Figure 12:**
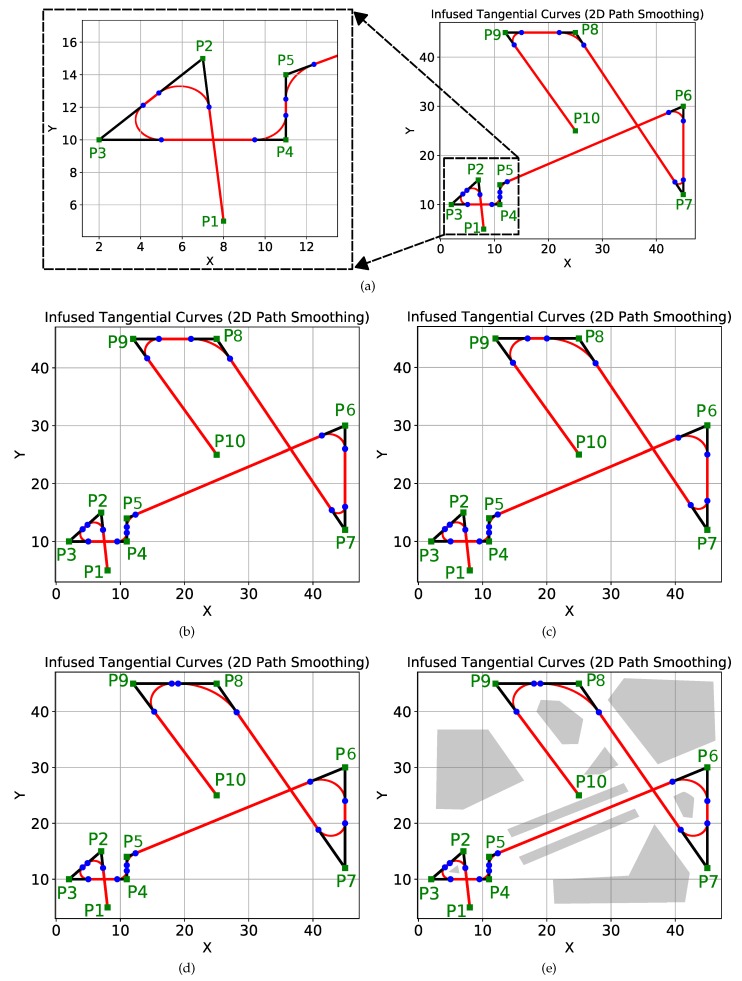
Comparison with interpolation based smoothing proposed in [56] using the same set of points. The various thresholds $\delta_{\mathrm{thresh}}$ in (**a**–**e**) are given in Table 5.

In the comparison, we used the same dataset of points as used in work [56]. The dataset contains total ten points in a grid of size 50×50 units. The 2D coordinates of the ten points have been summarized in Table 5. As shown in Figure 12, the ten points (P1,P2,⋯,P10) are marked in green. The starting point is P1:x=8,y=5, and the goal point is P10:x=25,y=25 with many sharp turns at different points. In Figure 12, the original path is marked in black, whereas the smooth path is shown in red.

To smooth the paths, we used four sets of thresholds. The results of smoothing with different thresholds are shown in Figure 12a–d. The values of different thresholds along with the 2D coordinates of the points are summarized in Table 5 for the different figures.

Figure 12a shows the path smoothing with a minimum threshold. The left section of the figure has been enlarged to provide a better view. We successively increased the clearance thresholds in different steps. The consequent smoothing results are shown in Figure 12b–d. Path smoothing at point P7 can be seen in different figures to notice the curvature change of the induced curve.

Figure 12e is the same as Figure 12d, but the obstacles are also shown. Notice that the path between the points P5 and P6 is a straight corridor between the walls shown in gray color. A robot traversing this path is expected to maintain a safe distance from the walls and move as straight as possible. The proposed method is able to achieve exactly that goal. The straight paths are kept straight, while only the sharp turns are smoothed out. For comparison, we direct the readers to paper [56] (page 7, Figure 11 of [56] to be exact). In work [56], since interpolation techniques are used, the path between the points P5 and P6 is not straight but dangerously close to the walls at multiple points. This also happens at other locations, and the robot’s proximity to the obstacles is compromising safety. Moreover, since interpolation is used, it is difficult to control the curvature of paths, especially at the turns. On the other hand, in the proposed method, it is easy to control the curvature.

However, our proposed method has a disadvantage that $G^{2}$ continuity is not possible. $G^{2}$ continuity is important for a robot that accelerates significantly on the paths. In fact, the reason why the work in [56] brings the robot close to one of the walls is because it emphasizes achieving a $G^{2}$ continuity. Adjusting a particular point on paths using interpolation is difficult as changing one point changes the whole path. Hence, there is a possibility that adjusting one point to a safe distance brings other portions of the continuous path close to the obstacles. The proposed method only guarantees a $G^{1}$ continuity that is tangential continuity. The $G^{1}$ continuity is important so that the robot does not experience a sudden kink or bump while traversing from a straight line to a curve. $G^{2}$ continuity is important for robots traveling at high speeds. However, most of the service robots have limited speed (generally around 2 m/s) to ensure operational safety. Although ensuring a $G^{2}$ continuity is beneficial, $G^{1}$ continuity is enough for operations at lower speeds. Since the proposed method only smooths the turns and keeps the straight paths straight, a robot can always navigate the straight segments of the path at high speeds and slow down before approaching a turn.

### 6.4. Generation of Smooth Paths with Variable Curvature

**Figure 13:**
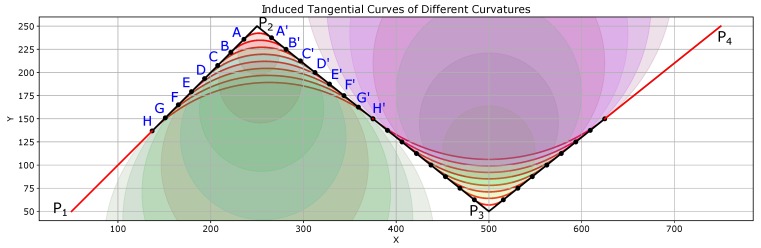
ITC path generation with different curvatures.

A strong merit of the proposed ITC path smoothing is that the curvature of the smooth trajectories can be controlled easily. Figure 13 shows the results of path smoothing in which the ITC curves have been generated with different curvatures. The original path is $P_{1}P_{2}P_{3}P_{4}$ shown in black color with sharp turns at points $P_{2}$ and $P_{3}$. The coordinates of the four points are: $P_{1}:\left(50,50\right),\;P_{2}:\left(250,250\right),\;P_{3}:\left(500,50\right),\;P_{4}:\left(750,250\right)$.

**Figure 14:**
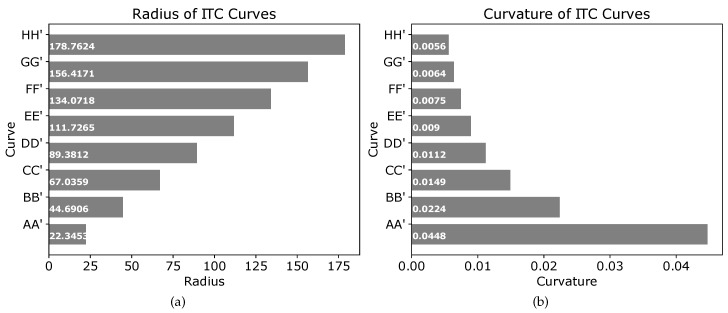
Radius and curvature of the different curves in Figure 13. (**a**) radius of curves at different points; (**b**) curvature of curves at different points.

As shown in Figure 13, at the same turn point $P_{2}$, different ITC curves ($\overset{⏜}{{\mathrm{AA}}^{\prime }}$, $\overset{⏜}{{\mathrm{BB}}^{\prime }}$, ⋯, $\overset{⏜}{{\mathrm{HH}}^{\prime }}$) have been generated. The radius and curvature of the different ITC curves are shown in Figure 14a,b, respectively. Thus, depending on the kinematics of the robot and the configuration of obstacles, appropriate path smoothing can easily be achieved using the proposed method.

### 6.5. Results of Smoothing in Real Environment with Actual Robots

**Figure 15:**
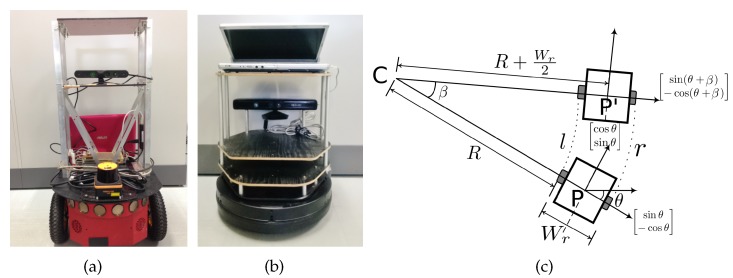
Robots used in the experiments. (**a**) Pioneer P3DX; (**b**) Kobuki Turtlebot; (**c**) Motion Model.

This section discusses results in a real environment with actual robots. Figure 15 shows the robots used and its motion model. We used a Pioneer-P3DX [61] robot and Turtlebot robot [62] shown in Figure 15a,b, respectively. Both the robots were equipped with distance sensors (Microsoft Kinect [63] and UHG-08LX laser range sensor [64]) and cameras. The distance sensor is accurate within ±30 mm within 1 m, and within 3% of the detected distance between 1 and 8 m. The angular resolution is approximately 0.36 degrees, and other specifications can be found in [64]. Specifications of Kinect sensors can be found in [63]. The robots were programmed in ROS [65]. Both are differential drive robots. We adopt the motion model from our previous work [52] and briefly describe here. The distance between the left and the right wheel is $W_{r}$, and the robot state at position *P*, is given as [x,y,θ]. From Figure 15c, turning angle β is calculated as

(35) $$\begin{array}{cc} r & =\beta \;\cdot \;(R+W_{r}),\\ l & =\beta \;\cdot \;R,\\ ∴\beta & =\frac{r-l}{W_{r}}, \end{array}$$

and the radius of turn *R* as

(36) $$\begin{array}{c} R=\frac{l}{\beta },\beta \neq 0. \end{array}$$

The coordinates of the center of rotation (*C*, in Figure 15c) are calculated as

(37) $$\left[\begin{array}{c} Cx\\ Cy \end{array}\right]=\left[\begin{array}{c} x\\ y \end{array}\right]-\left(R+\frac{W_{r}}{2}\right)\;\cdot \;\left[\begin{array}{c} \sin\theta \\ -\cos\theta \end{array}\right].$$

The new heading $\theta^{\prime }$ is

(38) $$\theta^{\prime }=\left(\theta +\beta \right)\;\mathrm{mod}\;2\pi ,$$

from which the coordinates of the new position $P^{\prime }$ are calculated as

(39) $$\left[\begin{array}{c} x^{\prime }\\ y^{\prime } \end{array}\right]=\left[\begin{array}{c} Cx\\ Cy \end{array}\right]-\left(R+\frac{W_{r}}{2}\right)\;\cdot \;\left[\begin{array}{c} \sin\theta^{\prime }\\ -\cos\theta^{\prime } \end{array}\right],\;\;\beta \neq 0\;\Rightarrow r\neq l.$$

If r=l, i.e., if the robot motion is straight, the state parameters are given as

(40) $$\theta^{\prime }=\theta ,$$

and

(41) $$\begin{array}{c} \left[\begin{array}{c} x^{\prime }\\ y^{\prime } \end{array}\right]=\left[\begin{array}{c} x\\ y \end{array}\right]+l\;\cdot \;\left[\begin{array}{c} \cos\theta \\ \sin\theta \end{array}\right],\;\left(l=r\right). \end{array}$$

**Figure 16:**
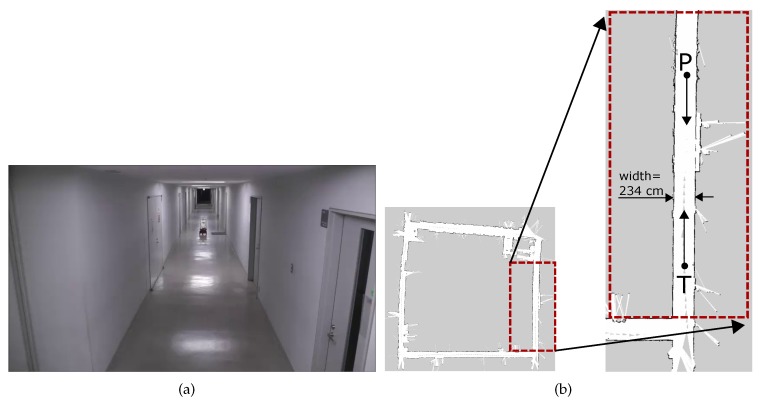
Experiment setup. (**a**) corridor scenario used in experiments; (**b**) grid-map of the environment. The zoomed out section shows the width of the corridor. Starting locations and directions of movement of the two robots are also shown.

Figure 16 shows the experiment environment and its grid map. As shown in Figure 16a, the environment was conducted in a narrow corridor of our university. The corresponding grid map is shown in Figure 16b, in which the actual experiment section of the corridor is marked and shown enlarged.

#### 6.5.1. Non-Smoothed Collision Avoidance and Navigation (Real Environment with Actual Robots)

Path smoothing in open and static environments can easily be demonstrated. However, real-time path smoothing for multiple robots in a dynamic and realistic scenarios is more challenging. We tested the proposed path smoothing method in a dynamic multi-robot collision avoidance scenario. In the experiment, the Pioneer P3DX robot and Turtlebot robot navigated towards each other in the narrow corridor and tried to avoid collision. We compared the trajectories in both traditional (non-smoothed) and the proposed (smoothed) method.

The direction of movement of both the robots is indicated in Figure 16b. Pioneer P3DX robot robot navigated from North to South direction with the starting point marked as P. The Turtlebot robot navigated in the opposite direction in the same corridor from South to North with the starting point marked as T in Figure 16b. The width of the corridor was 234 cm, and both the robots were programmed to navigate the center of the corridor (i.e., ≈ 127cm from either of the walls). The threshold collision avoidance distance was set to 4 m. Once an obstacle at this distance is found, the robots were programmed to avoid it using the traditional and proposed smooth algorithms.

**Figure 17:**
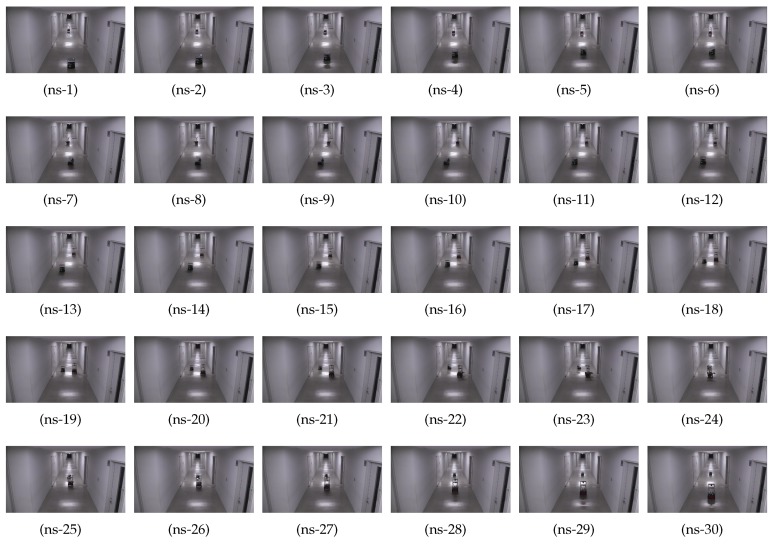
Timely snapshots of the non-smoothed navigation and collision avoidance. The robot actions at different steps are summarized in Table 6. Please see the supplementary video.

**Figure 18:**
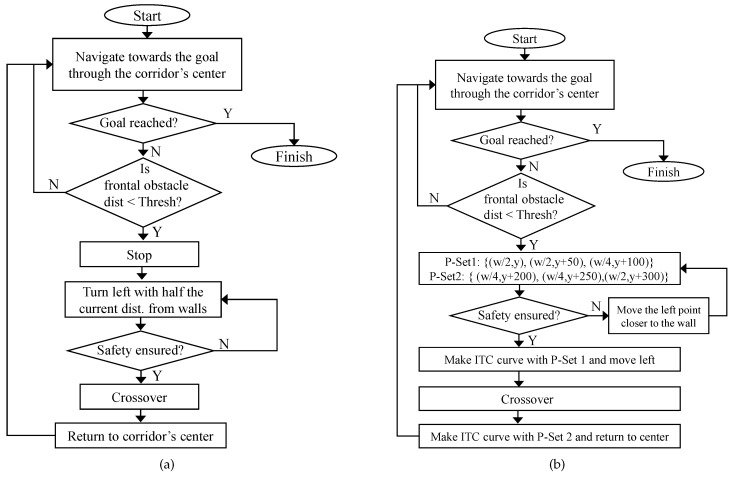
Flowchart of non-smoothed and ITC based smooth navigation and collision avoidance. (**a**) non-smoothed case; (**b**) ITC based smoothed case.

Figure 17 shows the timely snapshots (Figure 17(ns-1), ⋯, Figure 17(ns-30)) of the experiment with traditional path planning and navigation. For the ease of readability, we have summarized the various actions take by the two robots at different time-steps in Figure 17 in Table 6. The flowchart of the non-smoothed collision avoidance and navigation is shown in Figure 18a.

In the traditional navigation without smoothing, the robots stopped when the frontal distance was less than the threshold distance. Then, the robots took a sharp 90-degree turn towards the left, moved towards the wall, stopped, and again took a 90-degree right turn. The robots crossed-over, and then repeated the process to come to the center of the corridor from where they continued to navigate towards their respective goals.

Readers are advised to see the attached video to see the traditional navigation and multi-robot collision avoidance. It is clear that such a robot navigation with abrupt stops, and sharp turns, is not natural, potentially hazardous for the items carried on the robot, and even dangerous for people moving in the vicinity.

#### 6.5.2. Smoothed Collision Avoidance and Navigation (Real Environment with Actual Robots)

**Figure 19:**
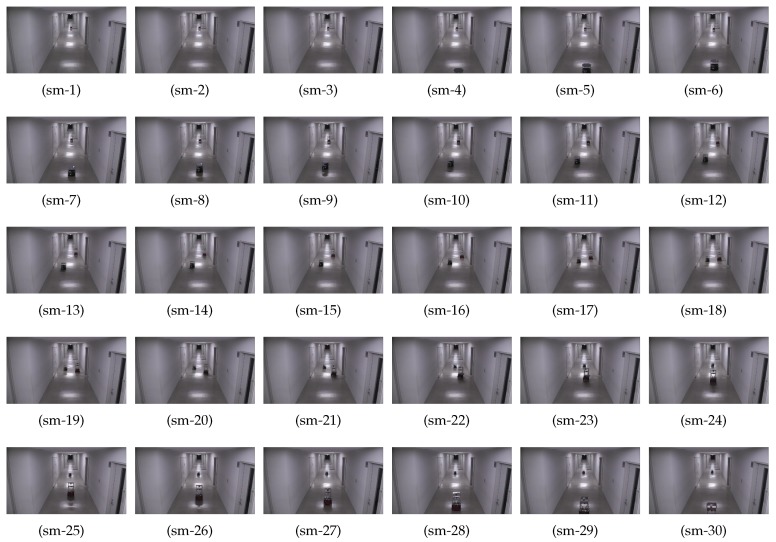
Timely snapshots of ITC based smoothed navigation and collision avoidance. The robot actions at different steps are summarized in Table 7. Please see the supplementary video.

Figure 19 shows the timely snapshots (Figure 19(sm-1), ⋯, Figure 19(sm-30)) of the navigation with the proposed smoothing algorithm. The experiment was conducted in the same environment with the same direction, start, and goal locations of Turtlebot and Pioneer robots. The flowchart of the smoothed collision avoidance and navigation is shown in Figure 18b.

The two robots approached each other while traversing the center of the corridor. When the frontal threshold distance was less than the threshold distance, the robots essentially generated three control points over which the tangential curve could be induced. As shown in Figure 18b, if *W* is the width of the corridor, the robot is traversing the corridor on a line $\left\{\frac{W}{2},y\right\},\;y\in I\;R_{\mathrm{map}}$ the robot generates a set (P-Set1) of three points:

(42) $$\text{P-Set}1=\left\{A:\left(\frac{W}{2},y\right),B:\left(\frac{W}{2},y+\psi_{1}\right),C:\left(\frac{W}{\lambda },y+2\psi_{2}\right)\right\}.$$

Essentially, the point $A=(\frac{W}{2},y)$ and $B=(\frac{W}{2},y+\psi_{1})$ lies on the straight line on the center of the corridor. Point $C=(\frac{W}{\lambda },y+2\psi_{2})$ lies on the left side of the corridor. The parameter λ controls the distance of the trajectory from the left wall of the corridor. For collision avoidance, λ is generally set to 4, which generates a trajectory at a distance of $\frac{W}{4}$ from the left wall of the corridor. The parameter $\psi_{1}$ controls the turning point in front of the robot. The parameter $\psi_{2}$ controls the point on the frontal left side of the robot. The effect of the parameter ψ is explained later. From the point set (P-Set1), an ITC curve is generated and induced in the original trajectory.

Once the robots have shifted left, they move in a straight line and cross each other. Once the robots have crossed over, the robots need to get back to the center of the corridor again. This is done by generating a set (P-Set2) of three points:

(43) $$\text{P-Set}2=\left\{A:\left(\frac{W}{\lambda },y_{t}\right),B:\left(\frac{W}{\lambda },y_{t}+\psi_{2}\right),C:\left(\frac{W}{2},y_{t}+2\psi_{2}\right)\right\}.$$

An ITC is generated again from set (P-Set2), and the robots smoothly traverse it to come back to the center of the corridor and navigate towards their respective goals.

We have summarized the various actions taken by the two robots at different time-steps in Figure 19 in Table 7.

**Figure 20:**
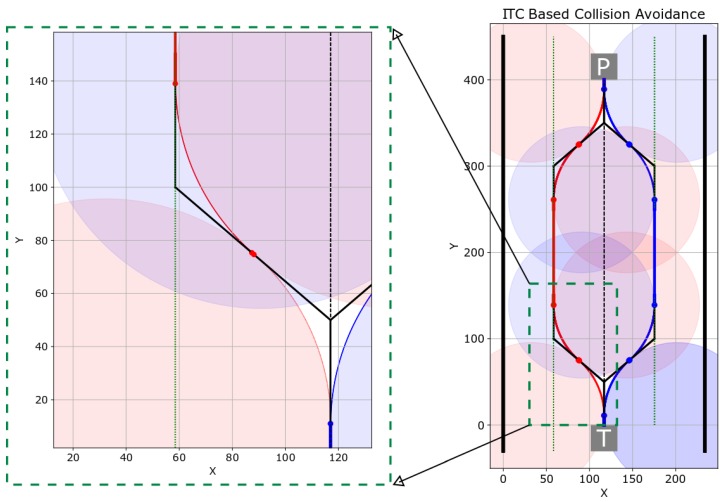
Trajectories of the two robots. Pioneer P3DX and Turtlebot are marked as P and T, respectively. The parameters were set as $\psi_{1}=\psi_{2}=50$.

Figure 20 shows the trajectories of the two robots. In Figure 20, ‘T’ and ‘P’ show the starting positions of Turtlebot and Pioneer robots, respectively. The trajectory of Turtlebot is shown in red, whereas the trajectory of Pioneer robot is shown in blue. The original trajectories are shown in black. As shown in Figure 16, the width of the corridor was 234 cm, and both the robots initially started from the center of the corridor. The center line is shown as a dotted line in Figure 20.

In the experiment shown in Figure 20, we set the parameters $\psi_{1}=\psi_{2}=50$. The Turtlebot’s starting position of turn was (x=117,y=0) and the Pioneer robot’s starting position at a smooth turn was (x=117,y=400). Setting the values in Equation (42), we get (P-Set1) as, $P-\mathrm{Set}1=\left\{A:(117,0),B:(117,50),C:(58.5,100)\right\}$. Similarly, another set of points are generated and the smooth ITC trajectories are generated for both Turtlebot (red trajectory in Figure 20) and Pioneer robot (blue trajectory in Figure 20). Figure 20 also shows a zoomed out section of the trajectory to visually confirm the induced $G^{1}$ tangential curves. In the experiment, the same values of both the parameters $\psi_{1}$ and $\psi_{2}$ were set for both of the robots. Hence, the generated trajectories in Figure 20 are symmetrical. The smooth ITC trajectories were generated in 39.73 ms on Ubuntu 16.04 with Core-i7 processor and 16 GB RAM using Python 3.6 language. This is fast enough for real-time applications.

### 6.6. Effect of ψ on a Smooth Trajectory

**Figure 21:**
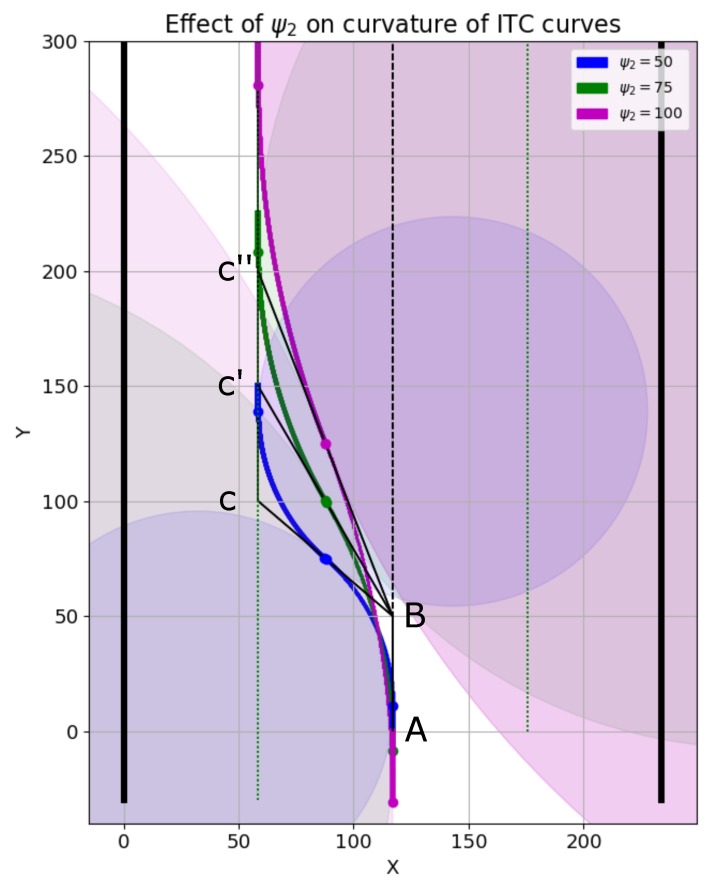
Effect of $\psi_{2}$ on ITC curve generation. Blue, green, and magenta curves were generated with $\psi_{2}=50$, $\psi_{2}=75$, and $\psi_{2}=100$, respectively.

We now discuss the effect of parameters $\psi_{1}$ and $\psi_{2}$ on the smoothness of ITC curves. The parameter $\psi_{1}$ controls the robot’s frontal starting point of trajectory generation. Parameter $\psi_{2}$ is crucial in controlling the curvature of the smooth trajectories. This is explained using Figure 21, in which the robot is assumed to be on the center of the corridor shown as a dotted line. Point A marks the starting of the smooth trajectory generation, and the second point B is at a distance of $\psi_{1}$ from point A. The points C, $C^{\prime }$, and $C^{\prime \prime }$ are generated using different values 50, 75, and 100 of $\psi_{2}$, respectively.

It should be noted that point B is common for the different ITC curves generated in Figure 21. However, the values of $\psi_{2}$ were different generating different set of points on the left side of the corridor. The blue ITC curve in Figure 21 corresponds to $\psi_{2}=50$. The green and magenta ITC curves corresponds to $\psi_{2}=75$, and $\psi_{2}=100$, respectively. The seed point set for blue ITC curve generation was $P-\mathrm{Set}1=\left\{A:(117,0),B:(117,50),C:(58.5,100)\right\}$. The seed point set for green ITC curve generation was: $P-\mathrm{Set}1=\left\{A:(117,0),B:(117,50),C:(58.5,125)\right\}$. Similarly, the seed point set for magenta ITC curve generation was: $P-\mathrm{Set}1=\left\{A:(117,0),B:(117,50),C:(58.5,150)\right\}$.

**Figure 22:**
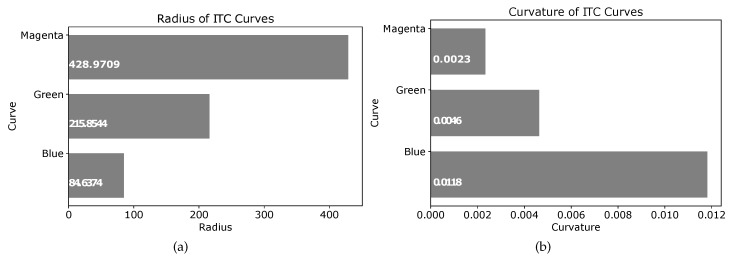
Radius and curvature of blue ($\psi_{2}=50$), green ($\psi_{2}=75$), and magenta ($\psi_{2}=100$) curves in Figure 21. (**a**) Radius of curves at different points. (**b**) Curvature of curves at different points.

The radius of the three curves seen in Figure 21 are shown in the plot of Figure 22a. Similarly, the curvatures of the three curves are given in Figure 22b. It is clear that increasing the value of $\psi_{2}$ generates an ITC curve with lesser curvature. The actual radii and curvatures of the three curves are shown in Figure 22a,b, respectively.

### 6.7. Effect of λ on Smooth Trajectory

**Figure 23:**
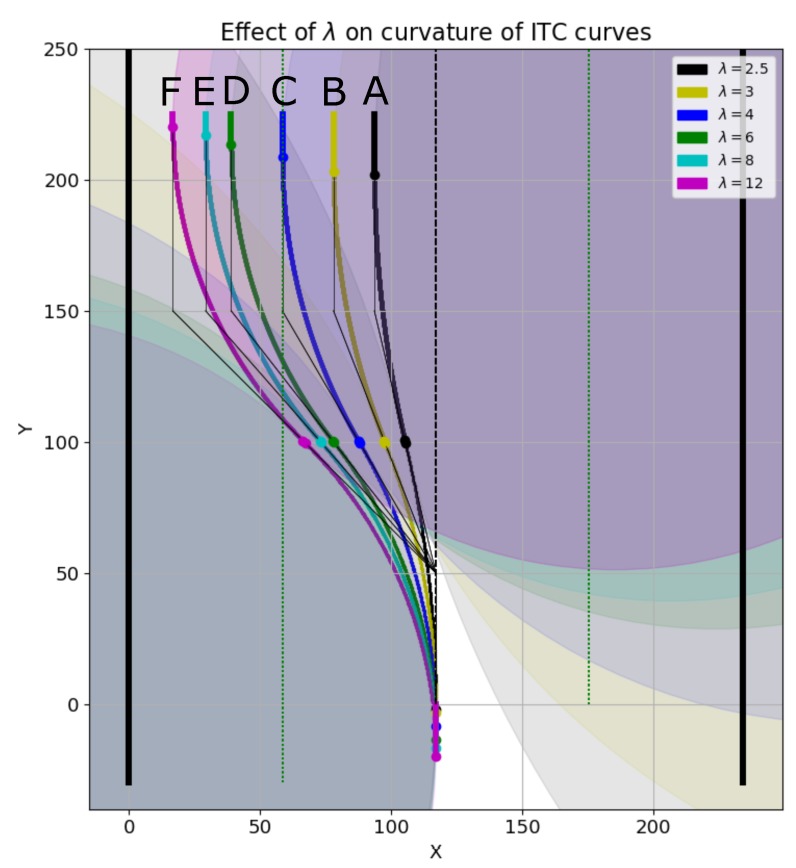
Effect of λ on different ITC curve generations for smooth left turns.

**Figure 24:**
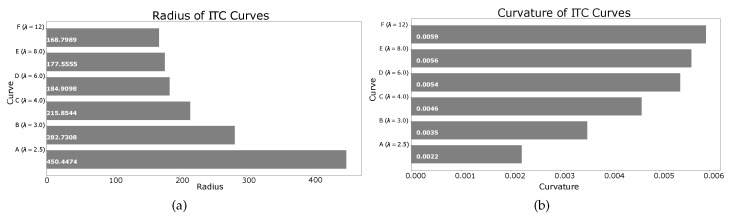
Radius and curvature of curves corresponding to different λ in Figure 23. (**a**) radius of curves at different points; (**b**) curvature of curves at different points.

The parameter λ in Equation (42) controls the distance of the trajectory from the left wall of the corridor. Figure 23 shows the smooth ITC trajectory generation with different values of λ. For generating the left turn, the condition is:

$$2<\lambda <\frac{W_{\mathrm{corridor}}}{\frac{W_{\mathrm{robot}}}{2}+\delta_{\mathrm{thresh}}},$$

where $W_{\mathrm{robot}}$ is the width of the robot, and $\delta_{\mathrm{thresh}}$ is the safety threshold from the corridor’s left wall. Setting λ=2 generates points on the straight line in the center of the corridor which does not require smoothing. As shown in Figure 23, the black ITC curve marked ‘A’ is generated with λ=2.5, and is closest to the corridor’s center. On the other hand, the magenta ITC curve marked ‘F’ is generated with λ=12, and is the farthest from the corridor’s center and closest to the corridor’s left wall. The other curves with different values of λ are also shown. The radii and curvatures of the different curves are shown in Figure 24a,b, respectively.

**Figure 25:**
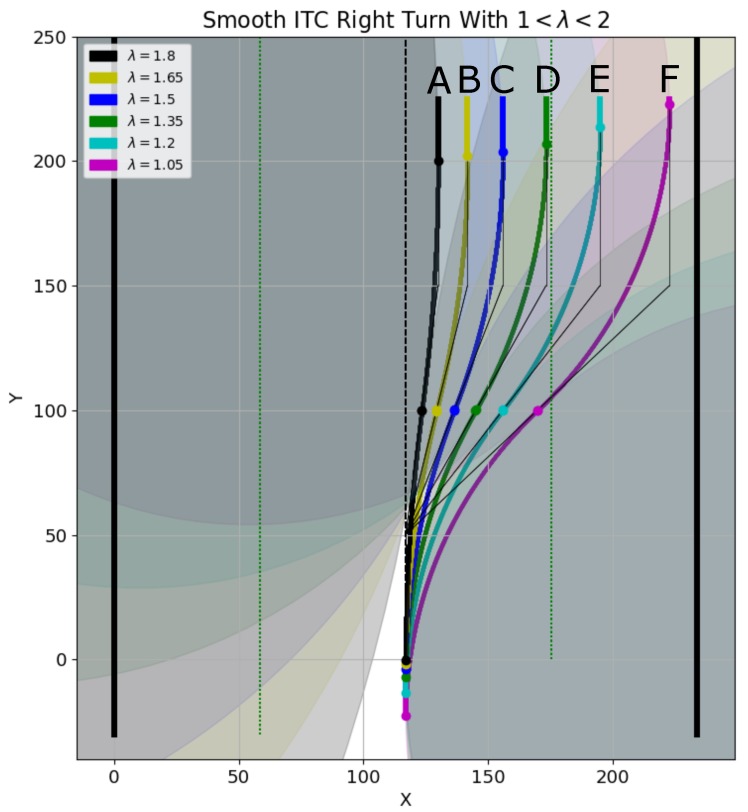
Effect of λ on different ITC curve generations for smooth right turns.

The same parameter is also used for generating the smooth right turn of the robot. Figure 25 shows right turn trajectory generation. The value of λ for right turn generation is

$$\frac{W_{\mathrm{corridor}}}{W_{\mathrm{corridor}}-\left(\frac{W_{\mathrm{robot}}}{2}+\delta_{\mathrm{thresh}}\right)}<\lambda <2.$$

**Figure 26:**
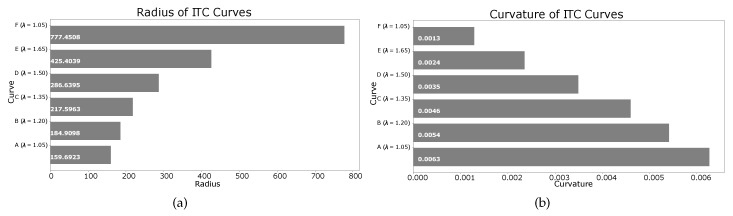
Radius and curvature of curves corresponding to different λ in Figure 25. (**a**) radius of curves at different points; (**b**) curvature of curves at different points.

As shown in Figure 25, the black ITC curve marked ‘A’ is generated with λ=1.8, and is closest to the corridor’s center. On the other hand, the magenta ITC curve marked ‘F’ is generated with λ=1.05, and is the farthest from corridor’s center, and closest to the corridor’s right wall. Setting λ=1 generates a trajectory touching the right wall of the corridor. The other curves with different values of λ are also shown. The radii and curvatures of the different curves are shown in Figure 26a,b, respectively.

Thus, depending on the width of the robot and the obstacle ahead, appropriate value of λ can be chosen to avoid collision, for both right and left turns.

## 7. ITC as a Path Smoothing Extension

**Figure 27:**
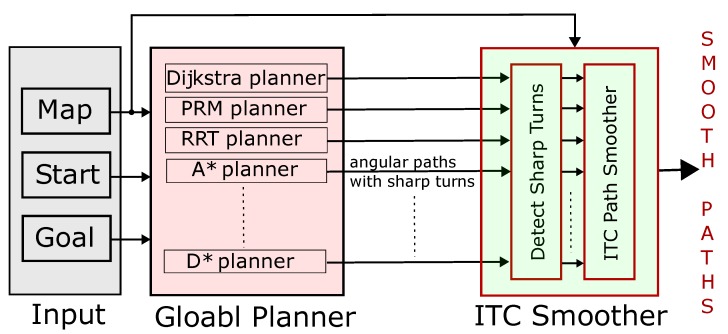
ITC path smoothing extension.

An ITC path smoother is proposed to work in conjunction with traditional path planning algorithms, and not to replace them. The overall idea of ITC as an ‘extension’ is shown in Figure 27. The input to the global path planner is: (a) map with obstacles and free space marked, (b) start, and (c) goal location in the map. Any of the global path planners like A*, D*, PRM, or RRT path planners can be used. The output of the global path planner is the input to the proposed ITC path smoother. The ITC smoother first detects the sharp turns and then smooths only the turns while keeping the straight segments straight. It can be seen in Figure 27 that the map information is also input to the ITC block. This is because ITC trajectories are generated keeping a safe distance from the obstacles. Thus, a map which marks the location of obstacles is required. The outputs of the ITC smoother are smooth trajectories whose angular turns have been smoothed out. In this way, the proposed ITC algorithm can be used as an extension with existing planners. A major benefit of such extension is that there is no need to replace the already tested planning algorithms used with the robots. The embedded software used in robot platforms is generally tightly coupled with the hardware and replacing the existing algorithms with new algorithms is generally avoided unless absolutely necessary as additional testing and benchmarking must be performed for the new algorithm. In this regard, the proposed ITC extension will integrate easily with existing algorithms. In addition, there is a lot of scope to customize the ITC smoother as generation of smooth trajectories is done on a part-by-part basis and there is much less computational overhead.

## 8. Conclusions

We presented a new algorithm called ITC for smooth trajectory generation for mobile robot robots. The algorithm can smooth out the sharp turns in the path generated by the global path planner. The trajectories generated by the algorithm are tangential to the path, thus preserving $G^{1}$ continuity. The curves can be generated fast in real-time by using only three key points on the path. Safety is embedded in an ITC algorithm, and it is guaranteed that the robot maintains a safe threshold distance from the obstacles, which is a crucial feature of mobile robot navigation. An essential feature of the algorithm is that only the turns are smoothed out, while the straight paths are kept straight. This feature is highly desired in case of mobile robot navigation in narrow corridors. We discussed ITC curve generation for both 2D path smoothing for UGVs, and 3D path smoothing for UAVs.

We compared the proposed ITC algorithm with interpolation based approaches. The comparison shows that ITC paths maintain a safe distance from the walls of the corridors and enables the robot to move in the center. Unlike interpolation algorithms, it is easier to define the control points in ITC algorithm. Moreover, unlike interpolation based methods, changing one point does not alter the whole path. This is another merit of the proposed ITC algorithm—that it is easy to define control points, and smoothing is done on a part-by-part basis for each turn. Thus, smoothing one section of the path with sharp turns does not affect other paths. It is difficult to follow such an approach in interpolation based algorithms as smoothing has to be done on a global basis, otherwise discontinuities or kinks get introduced in the overall path. The proposed ITC algorithm has a disadvantage that $G^{2}$ continuity is not guaranteed. This may be a limitation for mobile robots moving at high speed. However, for lower to medium speed robot navigation, this is not a problem. In fact, robots can traverse the straight sections of the ITC path with high speed, and slow down at the turns while executing a smooth turn, which is normally the case seen in robot’s navigation to ensure safety due to a change in view, and the sudden appearance of moving obstacles at turn points. We showed how ITC curves can be generated for different curvatures for smooth left and right turns by easily defining the parameters. Finally, we showed a complex real world scenario of collision avoidance with real robots. We compared the traditional navigation of the robots for collision avoidance with the proposed ITC based navigation and collision avoidance. It was clear that traditional navigation required the robot to stop and execute sharp turns. However, the ITC based navigation was smooth, natural, and robots could avoid collision by computing the smooth trajectories in real-time. Our current work presented a real-world 2D navigation of mobile robots and complex scenarios of collision avoidance. In the future, we will test the algorithm with multiple UAVs. A ground robot’s 2D navigation and collision avoidance will be compared to other algorithms with actual robots. In addition, apart from collision avoidance, we also consider trajectory smoothing of robotic arm manipulators as future work.

## Figures and Tables

**Table 1:** 2D coordinates of turn points in Figure 8a.

Coord	P-1	P-2	P-3	P-4	P-5	P-6	P-7	P-8	P-9	P-10	P-11	P-12	P-13	P-14
*X*	-2	2	4	7	8	16	10	11	17	19	13	13	18	23
*Y*	2	2	12	6	15	16	12	5	2	9	9	12	15	10
Threshold (δ): δ1 = 3, δ2 = 3, δ3 = 3, δ4 = 3, δ5 = 3, δ6 = 3, δ7 = 3, δ8 = 3, δ9 = 3, δ10 = 1, δ11 = 1.5, δ12 = 3														

**Table 2:** 3D coordinate of turn points in Figure 10a.

Coord	Point-1	Point-2	Point-3	Point-4	Point-5	Point-6	Point-7	Point-8	Point-9	Point-10
*X*	0	20	50	80	90	80	50	20	20	0
*Y*	0	10	20	20	50	80	80	60	40	0
*Z*	0	15	20	15	20	15	20	15	10	0
Threshold Distance (δ): δ1 = 14, δ2 = 13, δ3 = 14, δ4 = 15, δ5 = 12, δ6 = 12, δ7 = 10, δ8 = 9										

**Table 3:** 3D coordinate of turn points in Figure 10b.

Coord	Point-1	Point-2	Point-3	Point-4	Point-5	Point-6	Point-7	Point-8	Point-9	Point-10
*X*	0	60	80	90	70	60	50	30	20	0
*Y*	0	80	70	40	60	90	50	60	20	0
*Z*	0	85	25	55	20	35	60	15	10	0
Threshold Distance (δ): δ1 = 18, δ2 = 18, δ3 = 16, δ4 = 16, δ5 = 14, δ6 = 16, δ7 = 18, δ8 = 23										

**Table 4:** 3D coordinate of turn points in Figure 10c.

Coord	Point-1	Point-2	Point-3	Point-4	Point-5	Point-6	Point-7	Point-8
*X*	0	45	90	90	90	45	0	0
*Y*	0	0	0	45	90	90	90	45
*Z*	0	90	0	90	0	90	0	90
Threshold Distance (δ): δ1 = 35, δ2 = 35, δ3 = 35 δ4 = 35, δ5 = 35, δ6 = 35, δ7 = 35								

**Table 5:** Coordinates of different points in the comparative work of Figure 12 for different cases.

Coord	Point-1	Point-2	Point-3	Point-4	Point-5	Point-6	Point-7	Point-8	Point-9	Point-10
*X*	8	7	2	11	11	45	45	25	12	25
*Y*	5	15	10	10	14	30	12	45	45	25
Figure 12a Threshold Distance (δ): δ1 = 3, δ2 = 3, δ3 = 1.5, δ4 = 1.5, δ5 = 3, δ6 = 3, δ7 = 3, δ8 = 3										
Figure 12b Threshold Distance (δ): δ1 = 3, δ2 = 3, δ3 = 1.5, δ4 = 1.5, δ5 = 4, δ6 = 4, δ7 = 4, δ8 = 4										
Figure 12c Threshold Distance (δ): δ1 = 3, δ2 = 3, δ3 = 1.5, δ4 = 1.5, δ5 = 5, δ6 = 5, δ7 = 5, δ8 = 5										
Figure 12d Threshold Distance (δ): δ1 = 3, δ2 = 3, δ3 = 1.5, δ4 = 1.5, δ5 = 6, δ6 = 8, δ7 = 6, δ8 = 6										

**Table 6:** Description of robot actions at different steps (non-smooth case of Figure 17).

Figure	Action	Description
Figure 17(ns-1)∼(ns-4)	Move Straight	Robots navigate towards each other
Figure 17(ns-5)	Stop	Both the robots stop upon threshold distance
Figure 17(ns-6)∼(ns-9)	Move Left	Robots move left to ≈127 cm of the wall
Figure 17(ns-10)∼(ns-12)	Stop and Turn Right	Robots turn right
Figure 17(ns-13)∼(ns-20)	Crossover	Robots cross each other
Figure 17(ns-21)	Stop	Robots stop to make a turn again
Figure 17(ns-22)∼(ns-25)	Approach Center and Turn	Robots navigate to center of corridor and turn
Figure 17(ns-26)∼(ns-30)	Move Straight	Robots continue towards their goal

**Table 7:** Description of robot actions at different steps (smooth case of Figure 19).

Figure	Action	Description
Figure 19(sm-1)∼(sm-7)	Move Straight	Robots navigate towards each other
Figure 19(sm-8)∼(sm-11)	Smooth Turn Left	Both the robots smoothly move to left
Figure 19(sm-12)∼(sm-20)	Crossover	Robots cross each other
Figure 19(sm-21)∼(sm-24)	Smooth Turn Right	Both the robots smoothly approach corridor’s center
Figure 19(sm-25)∼(sm-30)	Move Straight	Robot navigate towards their goal

## Supplementary Materials

Experiment video is available online at https://www.mdpi.com/1424-8220/19/20/4384/s1, Video S1: Video of Traditional Non-Smooth vs ITC Based Smooth Collision Avoidance of Multiple Robots.
